# Current and prospective roles of magnetic resonance imaging in mild traumatic brain injury

**DOI:** 10.1093/braincomms/fcaf120

**Published:** 2025-03-25

**Authors:** Matilde Sassani, Tara Ghafari, Pradeepa R W Arachchige, Iman Idrees, Yidian Gao, Alice Waitt, Samuel R C Weaver, Ali Mazaheri, Hannah S Lyons, Olivia Grech, Mark Thaller, Caroline Witton, Andrew P Bagshaw, Martin Wilson, Hyojin Park, Matthew Brookes, Jan Novak, Susan P Mollan, Lisa J Hill, Samuel J E Lucas, James L Mitchell, the UK mTBI Predict Consortium, Alexandra J Sinclair, Aliza Finch, Adam Hampshire, Alice Sitch, Ali Mazaheri, Andrew P Bagshaw, Asha Strom, Alice Waitt, Andreas Yiangou, Alexander Bennett, Angus Hunter, Caroline Witton, Davinia Fernández-Espejo, Dan Ford, Duncan Wilson, Hamid Dehghani, Hyojin Park, Hannah S Lyons, Helen Brunger, Henrietta Ellis, Iman Idrees, Ian Varley, Jessica Hubbard, Jun Cao, Jon Deeks, James L Mitchell, Jan Novak, Jamie Pringle, John Terry, Jack Rogers, Jessikah Fildes, Karen Mullinger, Lisa J Hill, Mark Thaller, Martin Wilson, Matilde Sassani, Matthew Brookes, Ned Jenkinson, Ole Jensen, Pete Hellyer, Sebastian Coleman, Raymond Reynolds, Richard Blanch, Katie Morris, Ryan Ottridge, Rachel Upthegrove, Pradeepa R W Arachchige, Sarah Berhane, Samuel J E Lucas, Sophie Prosser, Shreshth Dharm-Datta, Tara Ghafari, Waheeda Hawa, Yidian Gao, Alexandra J Sinclair, Karen Mullinger, Davinia Fernández-Espejo

**Affiliations:** Department of Metabolism and Systems Science, College of Medicine and Health, University of Birmingham, Birmingham B15 2TT, UK; Centre for Endocrinology, Diabetes and Metabolism, Birmingham Health Partners, Birmingham B15 2TH, UK; Department of Neurology, Queen Elizabeth Hospital, University Hospitals Birmingham NHS Foundation Trust, Birmingham B15 2WB, UK; Centre for Human Brain Health and School of Psychology, University of Birmingham, Birmingham B15 2TT, UK; Sir Peter Mansfield Imaging Centre, School of Physics and Astronomy, University of Nottingham, Nottingham NG7 2RD, UK; College of Health and Life Sciences, Aston Institute of Health and Neurodevelopment, Aston University, Birmingham B4 7ET, UK; Centre for Human Brain Health and School of Psychology, University of Birmingham, Birmingham B15 2TT, UK; Centre for Human Brain Health and School of Psychology, University of Birmingham, Birmingham B15 2TT, UK; College of Health and Life Sciences, Aston Institute of Health and Neurodevelopment, Aston University, Birmingham B4 7ET, UK; Centre for Human Brain Health and School of Sport, Exercise and Rehabilitation Sciences, University of Birmingham, Birmingham B15 2TT, UK; Centre for Human Brain Health and School of Psychology, University of Birmingham, Birmingham B15 2TT, UK; Department of Metabolism and Systems Science, College of Medicine and Health, University of Birmingham, Birmingham B15 2TT, UK; Centre for Endocrinology, Diabetes and Metabolism, Birmingham Health Partners, Birmingham B15 2TH, UK; Department of Neurology, Queen Elizabeth Hospital, University Hospitals Birmingham NHS Foundation Trust, Birmingham B15 2WB, UK; Department of Metabolism and Systems Science, College of Medicine and Health, University of Birmingham, Birmingham B15 2TT, UK; Centre for Endocrinology, Diabetes and Metabolism, Birmingham Health Partners, Birmingham B15 2TH, UK; Department of Metabolism and Systems Science, College of Medicine and Health, University of Birmingham, Birmingham B15 2TT, UK; Centre for Endocrinology, Diabetes and Metabolism, Birmingham Health Partners, Birmingham B15 2TH, UK; Department of Neurology, Queen Elizabeth Hospital, University Hospitals Birmingham NHS Foundation Trust, Birmingham B15 2WB, UK; College of Health and Life Sciences, Aston Institute of Health and Neurodevelopment, Aston University, Birmingham B4 7ET, UK; Centre for Human Brain Health and School of Psychology, University of Birmingham, Birmingham B15 2TT, UK; Centre for Human Brain Health and School of Psychology, University of Birmingham, Birmingham B15 2TT, UK; Centre for Human Brain Health and School of Psychology, University of Birmingham, Birmingham B15 2TT, UK; Sir Peter Mansfield Imaging Centre, School of Physics and Astronomy, University of Nottingham, Nottingham NG7 2RD, UK; College of Health and Life Sciences, Aston Institute of Health and Neurodevelopment, Aston University, Birmingham B4 7ET, UK; Department of Metabolism and Systems Science, College of Medicine and Health, University of Birmingham, Birmingham B15 2TT, UK; Birmingham Neuro-ophthalmology, Queen Elizabeth Hospital, University Hospitals Birmingham NHS Foundation Trust Birmingham, Birmingham B15 2WB, UK; Department of Biomedical Sciences, School of Infection, Inflammation and Immunology, College of Medicine and Health, University of Birmingham, Birmingham B15 2TT, UK; Centre for Human Brain Health and School of Sport, Exercise and Rehabilitation Sciences, University of Birmingham, Birmingham B15 2TT, UK; Department of Metabolism and Systems Science, College of Medicine and Health, University of Birmingham, Birmingham B15 2TT, UK; Centre for Endocrinology, Diabetes and Metabolism, Birmingham Health Partners, Birmingham B15 2TH, UK; Department of Neurology, Queen Elizabeth Hospital, University Hospitals Birmingham NHS Foundation Trust, Birmingham B15 2WB, UK; Department of Metabolism and Systems Science, College of Medicine and Health, University of Birmingham, Birmingham B15 2TT, UK; Centre for Endocrinology, Diabetes and Metabolism, Birmingham Health Partners, Birmingham B15 2TH, UK; Department of Neurology, Queen Elizabeth Hospital, University Hospitals Birmingham NHS Foundation Trust, Birmingham B15 2WB, UK; Centre for Human Brain Health and School of Psychology, University of Birmingham, Birmingham B15 2TT, UK; Sir Peter Mansfield Imaging Centre, School of Physics and Astronomy, University of Nottingham, Nottingham NG7 2RD, UK; Centre for Human Brain Health and School of Psychology, University of Birmingham, Birmingham B15 2TT, UK

**Keywords:** mild traumatic brain injury, concussion, biomarkers, magnetic resonance imaging, magnetic resonance spectroscopy

## Abstract

There is unmet clinical need for biomarkers to predict recovery or the development of long-term sequelae of mild traumatic brain injury, a highly prevalent condition causing a constellation of disabling symptoms. A substantial proportion of patients live with long-lasting sequelae affecting their quality of life and ability to work. At present, symptoms can be assessed through clinical tests; however, there are no imaging or laboratory tests fully reflective of pathophysiology routinely used by clinicians to characterize post-concussive symptoms. Magnetic resonance imaging has potential to link subtle pathophysiological alterations to clinical outcomes. Here, we review the state of the art of MRI research in adults with mild traumatic brain injury and provide recommendations to facilitate transition into clinical practice. Studies utilizing MRI can inform on pathophysiology of mild traumatic brain injury. They suggest presence of early cytotoxic and vasogenic oedema. They also show that mild traumatic brain injury results in cellular injury and microbleeds affecting the integrity of myelin and white matter tracts, all processes that appear to induce delayed vascular reactions and functional changes. Crucially, correlates between MRI parameters and post-concussive symptoms are emerging. Clinical sequences such as T_1_-weighted MRI, susceptibility-weighted MRI or fluid attenuation inversion recovery could be easily implementable in clinical practice, but are not sufficient, in isolation for prognostication. Diffusion sequences have shown promises and, although in need of analysis standardization, are a research priority. Lastly, arterial spin labelling is emerging as a high-utility research as it could become useful to assess delayed neurovascular response and possible long-term symptoms.

## Teaser

Sassani *et al*. review the current and prospective roles of magnetic resonance imaging in mild traumatic brain injury (mTBI), elucidating mTBI pathophysiology and highlighting potential of MRI as a biomarker in mTBI.

## Graphical Abstract

**Graphical Abstract:**
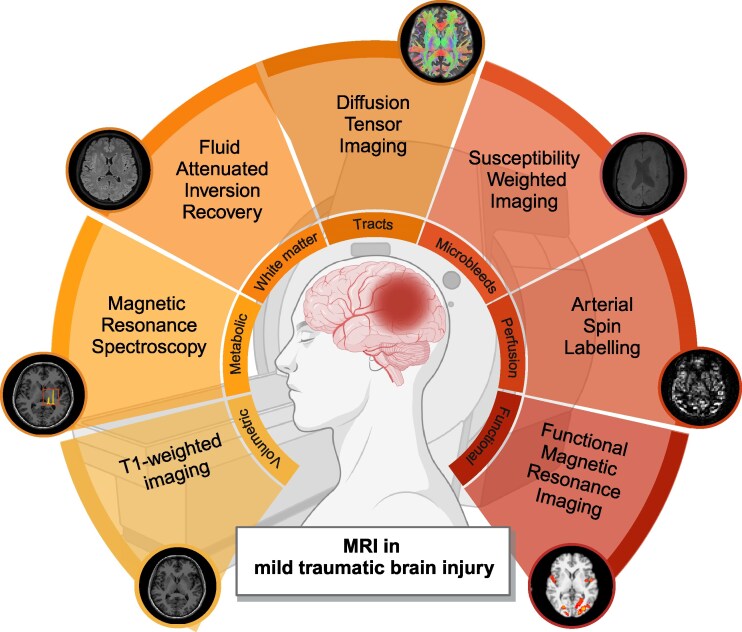
Created in BioRender. Sassani, M. (2025) https://BioRender.com/f37k702.

## Introduction

Traumatic brain injury (TBI) is a silent epidemic, estimated to affect ∼70 million people worldwide every year, with low- and middle-income countries carrying the greatest burden of disease.^1-3^ Mild traumatic brain injury (mTBI) accounts for between 70 and 90% of all brain injuries; disproportionately affecting teenagers and young adults^4^; and being prevalent amongst victims of domestic violence,^5^ those playing contact sports,^6^ people in correction and detention centres,^7^ active duty military personnel and veterans^8^ and those who are homeless.^9^

mTBI is caused by a traumatic event transmitting energy to the brain and resulting in transitory alterations in cerebral function. The term mTBI and concussion are often used interchangeably, although technically they are not synonyms, with concussion generally being considered a form of mTBI.^10^ The diagnosis of mTBI is clinical and, according to the ‘Mayo Classification System’, requires loss of consciousness lasting no more than 30 min, peri-injury amnesia of <24 h and a Glasgow coma scale score 13–15 after 30 min and, typically, no evidence of haemorrhage, brainstem injury, penetrating wound to the brain or contusion.^11^ Other analogous diagnostic criteria, such as those by the ‘World Health Organization’^12^ or the ‘American Congress of Rehabilitation’ Medicine,^13^ are also frequently used. Upon assessment, there are no focal neurological symptoms and standard clinical imaging, such as CT and anatomical MRI, often does not detect any visible pathology. Due to accessibility in the emergency department, CT is often the modality of choice, although expert opinion suggests its use is not always indicated in mTBI.^14,15^ In the research setting, CT imaging is rarely used. It has, however, been employed in 48 patients 1-year post-mTBI, and it showed some degree of global atrophy on a group level.^16^

Despite lack of focal neurological findings and objective confirmatory diagnostic tests, symptoms associated with mTBI can be pervasive and debilitating. The majority (up to over 90% in some studies) of patients report post-traumatic migrainous or tension-type headaches,^17-19^ often in conjunction with numerous somatic, cognitive, behavioural and affective symptoms.^20,21^ The constellation of post-injury symptoms is considerable and includes: blurred vision, diplopia, photopsia, photophobia and phonophobia, tinnitus, nystagmus, vertigo, nausea, vomiting, imbalance and dizziness.^22-25^ Patients can also suffer with difficulty concentrating and remembering, sleep disturbances, lethargy, being generally slower or having ‘brain fog’, emotional lability, anxiety, depression, irritability and/or fatigue.^26-30^ One of the challenges is to characterize objectively such symptoms that are often very disabling to patients, yet not captured well by available standardized tests. In addition, such symptoms may overlap and coexist with those of post-traumatic stress disorder (PTSD) and differential diagnosis can be challenging at times.^18,31^ Pre-injury mental health has been also shown to affect recovery.^32^

In adults, cognitive dysfunction and other self-reported symptoms tend to resolve within a few months. However, a significant proportion (∼30–40% in some studies) develop long-term sequelae, previously known as post-concussion syndrome, including poor cognitive outcome and injury-related life difficulties.^26,32-35^ Such long-term symptoms can impact quality of life, the ability of patients to return to work and to other pre-injury activities^36^ as well as having significant medico-legal implications.^37,38^ Hence, numerous patients require a detailed assessment of such long-term conditions and the efficacy of tailored rehabilitation strategies is being investigated.^39^ Head injury has also been associated with increased risk of developing psychiatric diseases, chronic traumatic encephalopathy and, possibly, neurodegenerative conditions such as Alzheimer's disease and motor neuron disease.^6,40-42^

At present, there are no reliable prognostic biomarkers used in clinical practice that can predict the development of long-term sequelae. In particular, there are no objective quantitative measures of disease that are specific and sensitive to mTBI and that correlate strongly with symptomatology. This is, at least partially, due to incomplete understanding of pathophysiology. Brain imaging modalities, such as MRI, are well placed to elucidate pathophysiology and might therefore be developed as diagnostic and prognostic tools.

**Figure 1:**
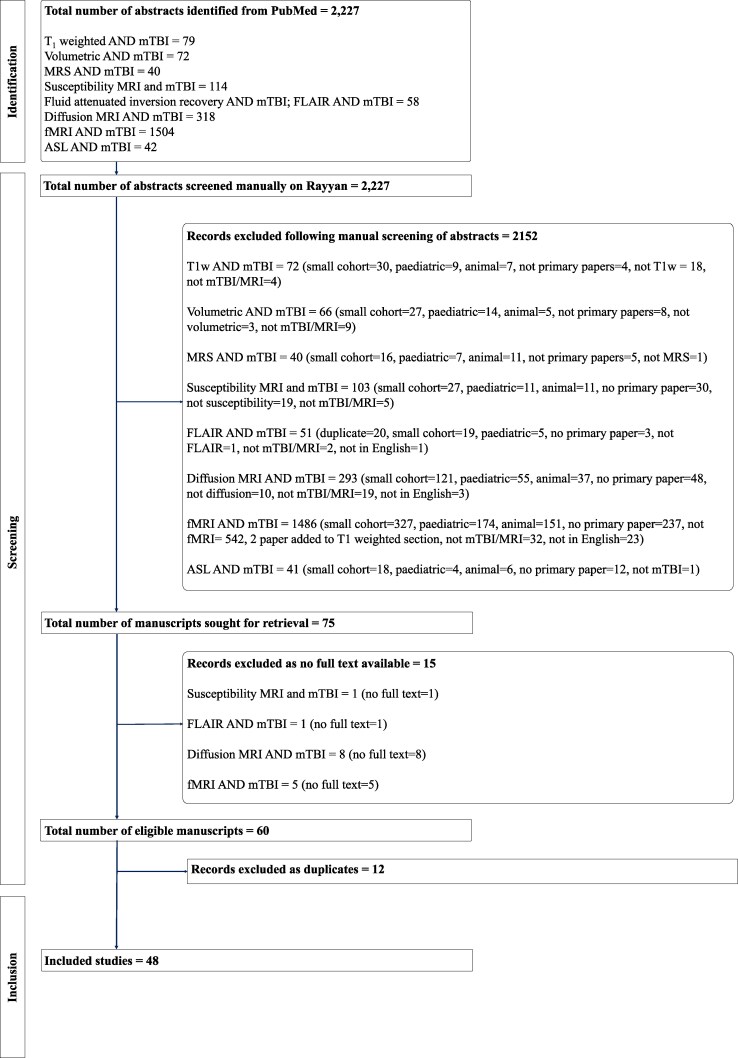
**Flow diagram illustrating identification, screening and selection of manuscripts included in the systematic search.** T1w, T_1_-weighted.

**Figure 2:**
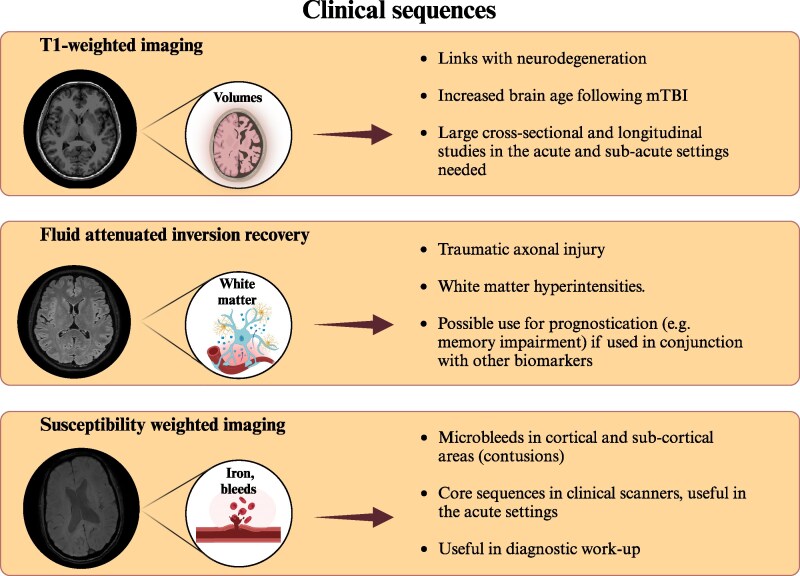
**Key highlights: sequences already widely used in clinical practice.** Created in BioRender. Sassani, M. (2025) https://BioRender.com/f5mhnnn.

**Figure 3:**
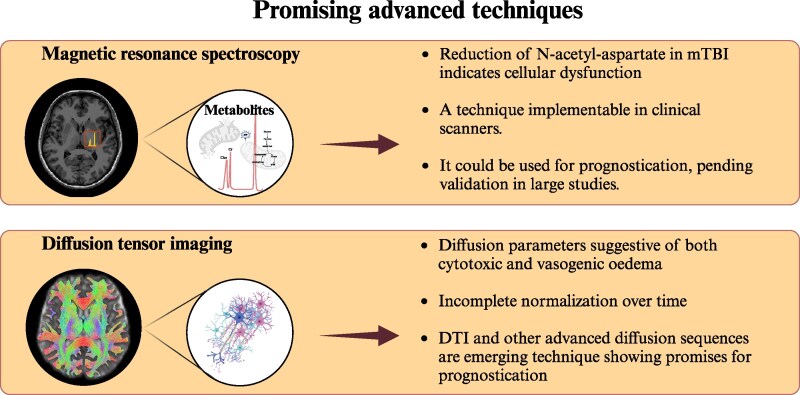
**Key highlights: magnetic resonance spectroscopy and DTI.** α-KG, alpha-ketoglutarate; ATP, adenosine triphosphate; Cho, choline; Co-A, coenzyme A; Cr, creatine. Created in BioRender. Sassani, M. (2025) https://BioRender.com/s04q864

**Figure 4:**
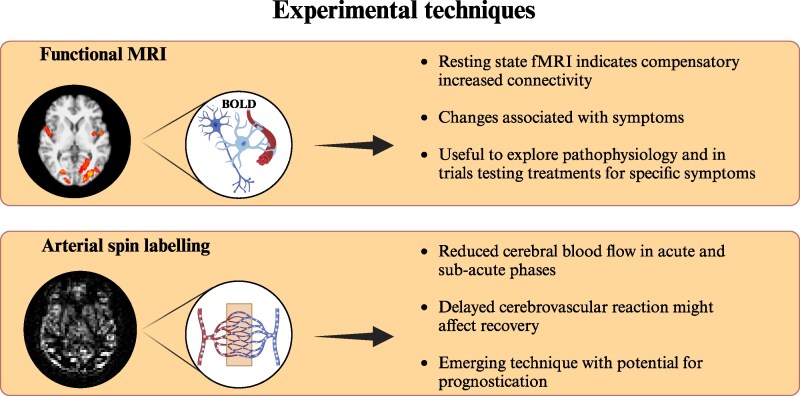
**Key highlights: experimental magnetic resonance imaging techniques.** BOLD, blood-oxygenation-level-dependent effect. Created in BioRender. Sassani, M. (2025) https://BioRender.com/29kenoz

This is a comprehensive review following systematic searches^43^ which aims to summarize and appraise the current evidence emerging from utilization of advanced MRI in adults with mTBI. The following MRI methods were assessed: T_1_-weighted magnetic resonance imaging, magnetic resonance spectroscopy, susceptibility-weighted magnetic resonance imaging (SWI), fluid-attenuated inversion recovery (FLAIR), diffusion-weighted imaging, functional magnetic resonance imaging (fMRI) and arterial spin labelling (ASL). Our main outcomes of interest were between-group differences in MRI parameters between mTBI patients and controls, presence of longitudinal changes in MRI parameters and association between MRI parameters and clinical measures. Firstly, we screened PubMed database for recent (i.e. published since 2020) systematic reviews appraising mTBI in each specific MRI methodology. Three systematic reviews were published recently: one in MRS and mTBI,^44^ one in diffusion imaging and mTBI^45^ and one in ASL and mTBI.^46^ For those techniques, we conducted systematic searches on PubMed to identify papers published after those included in the systematic reviews until 19 December 2023. For the other techniques where no recent systematic review was identified, a systematic search was conducted on PubMed for all papers from inception till 19 December 2023. Search terms are listed in the Supplementary Appendix 1. All selected results were uploaded onto the software rayyan (https://www.rayyan.ai/) where abstracts were screened by three independent and blinded reviewers (M.S. screened all 2227 abstracts, T.G. acted as the second blinded reviewer and screened first 1114 abstracts, and A.W. acted as the second blinded reviewer and screened the remaining 1113 abstracts). Articles were selected if they were primary research articles assessing mTBI and the technique of interest, if they were in English, in humans, had adult participants, had a cohort of mTBI encompassing at least 88 participants (this cohort size being derived from a G-power calculation for standard case–control studies assessing difference between the means to return 0.95 power), and if full text was available. After screening was completed, researchers were unblinded and any discrepancy resolved by consensus. A total of 2227 abstracts were screened and 48 articles selected. A flow diagram is reported in Fig. 1. For each selected study, the following data were extracted: number of mTBI and control participants, whether participants were military, civilians and/or sportsmen, type of injury, age range of inclusion criteria, mean age for mTBI cohort, number of men and female participants included with mTBI, timing of MRI scan post-injury, details of utilized MRI sequences and strength of magnet, main significant results and main significant correlates with clinical measures. A section on possible bias and limitations of the study was also included. The results are summarized in Supplementary Tables 1–3 and presented in the section below in a narrative manner. Figures 2–4 were created with BioRender.com, partially utilizing some BioRender templates (https://app.biorender.com/biorender-templates.&apos).

## Main body

Magnetic resonance imaging is not routinely used in the acute clinical management of mTBI due to possible contraindications, cost and time in scanner. In the acute settings, CT is widely regarded as sufficient for triage and identification of possible traumatic mass lesions and/or haemorrhages which might require surgical decompression or evacuation.^47^ For example, in the UK, the ‘National Institute for Health and Care Excellence’ guidelines do not recommend using MRI as primary investigation, but recognize the need to conduct further research as neuroimaging might be valuable for prognostication.^48^ On clinical scans, presence of cavum septum pellucidum^49^ or of haemorrhages^50^ can be visually detected in a proportion of patients. These patients might be classified as having mTBI or not depending on which diagnostic criteria are used. Regardless of classification, it is still debated whether presence of such changes is predictive of symptoms or of outcome with some studies having shown some predictive value,^51^ whereas others have not.^52,53^ Interestingly, presence of changes on CT does not correlate with MRI volumetric and morphological changes.^54^

### T_1_-weighted magnetic resonance imaging

Quantitative analyses of T_1_-weighted volumetric MRI as well as surface analyses have been used extensively in mTBI research. Volumetric studies with relatively small cohorts detected mild reductions in global and cortical volumes with enlargement of the ventricular system.^55-58^ Others have suggested that frontotemporal cortical areas are more prone to damage from traumatic injury and, hence, more likely to show focal volumetric atrophy^59^ and that volumes of hippocampus,^60^ thalamus^61,62^ and brainstem^63^ may also be reduced.

Interestingly, our systematic search of studies conducted in larger cohorts painted a different picture in suggesting no changes across a number of structural measures when comparing mTBI and healthy controls. Specifically, a study comparing 89 military with mTBI to 34 military controls 4 years post-injury found no significant differences in cortical thickness.^64^ Similarly, another large research in both military and civilian participants found no significant differences in 147 patients compared with 131 controls 7.5 years post-injury.^65^ Another study looking at preliminary data of a multi-site military consortium did not find any significant between-group differences in region of interest analysis in 269 mTBI compared with 50 controls; the study was conducted 8.8 years following index injury.^66^ Lastly, no between-group changes in whole brain and cortical grey matter volumes were detected in 171 veterans 20 years following injury compared with 115 veteran controls.^67^ Notably, all these larger studies assessed volumes cross-sectionally and many years following injury, and, to our knowledge, there is no large publication assessing volumetric changes longitudinally in the acute and sub-acute phases. It is therefore possible that putative volumetric changes may normalize over the years and that discrepancies in findings may be partially attributed to timing of imaging.

Some of the changes on T_1_-weighted imaging correlate with symptomatology on group-level analyses. For example, a large study in 89 patients scanned 2 weeks post-injury revealed significant correlates between cortical thinning and anxiety and depression, as well as between sub-cortical volumes and symptoms of somatization.^68^ There have also been attempts to utilize T_1_-weighted volumetric acquisitions to link mTBI and pathophysiology of Alzheimer's disease. One large study assessing putative effects of mTBI on neurodegeneration showed links between cortical thinning in posterior cingulate cortex and mTBI, mediated by polygenic risk score for developing Alzheimer's disease.^69^ Interestingly, these effects were independent of apolipoprotein E ɛ4 genetic status.^69^

Despite some evidence for clinical correlates and links with neurodegeneration, our systematic search revealed only two large studies that have assessed predictive power of volumetric parameters in mTBI. An earlier one in 147 patients demonstrated that incorporation of MRI acquired a month post-injury did not improve clinical predictive models.^70^ In contrast, Stein *et al.* utilized volume of superior frontal and anterior cingulate cortices in conjunction with numerous other clinical parameters to predict development of PTSD at 3 months. However, the model was not predictive of PTSD at 6 months,^71^ indicating that volumetric parameters may not be useful for long-term prognostication in clinical settings.

Specific analyses of T_1_-weighted volumetric acquisitions are increasingly being utilized to estimate brain age in mTBI. Our systematic search highlighted five large studies assessing brain age in mTBI. They consistently showed that predicted brain age in mTBI is elevated compared with patients’ chronological age and to controls’ brain age.^64,72-74^ The effect of mTBI on brain age has been shown to be more pronounced in those above 40 compared with younger patients,^73^ not to progress longitudinally^72,73^ and to be driven by specific brain regions: superior and middle frontal gyri, middle temporal gyrus, posterior dorsal cingulate gyrus and orbital sulci.^74^ Interestingly, predicted brain age in mTBI does not correlate with severity of post-concussive depression and PTSD.^64^ A study assessing 123 mTBI patients found that brain age gap was not significantly different in apolipoprotein E ɛ4 carriers compared with non-carriers,^75^ indicating that accelerated ageing is not increased in those at higher risk of Alzheimer's disease carrying apolipoprotein E ɛ4.

In summary, T_1_-weighted MRI has helped corroborating effects of mTBI on brain ageing, but its predictive power in mTBI is likely to be very limited. In addition, the current literature from large cross-sectional studies indicates lack of evidence for a significant reduction in volumetric brain parameters in mTBI many years post-injury. Large studies investigating volumetric changes longitudinally in the acute and sub-acute phases are an area of need in the field.

### Magnetic resonance spectroscopy

Magnetic resonance spectroscopy allows direct detection and quantification of metabolites *in vivo*. Three brain metabolites are consistently resolved by proton spectroscopy (^1^H-MRS): N-acetyl-aspartate (NAA), which is the most abundant cerebral metabolite; choline, a glial marker; and total creatine, which plays a role in the creatine kinase shuttle and, hence, mitochondrial bioenergetics. Specific sequences allow detection of other neurochemicals such as the neurotransmitter glutamate, the amino acid glutamine and the glial marker myoinositol.^76^

A recent systematic review synthetized and appraised available studies employing ^1^H-MRS in mTBI. Studies appraised reported metabolites from numerous anatomical areas using different sequences; nonetheless, the authors were able to conduct a meta-analysis as well.^44^ There was a consistent NAA reduction in corpus callosum as well as frontal, parietal and occipital lobes.^44,77^ Although the meta-analysis showed that creatine, choline, glutamate, glutamine and myoinositol did not have a significant effect size in mTBI,^44^ there is some early evidence suggesting that myoinositol, glutamate and glutamine are raised in putamen of patients, potentially indicating a reactive glial response and neuroexcitatory reaction.^78^

Joyce *et al.* also conducted a moderator analysis of longitudinal spectroscopic data in TBI. N-acetyl-aspartate reduction was more prominent in the hyper-acute phases following injury, although it was also detected in acute, sub-acute and chronic (i.e. more than 3 months) stages.^44^ White matter choline and creatine levels have been shown to have a positive association with time elapsed since injury.^79^

Sex had no impact on any of the resolved metabolites, whereas NAA, choline, glutamate, glutamine and myoinositol values were modulated by age.^44^ Choline and myoinositol were significantly affected by severity of injury with mTBI studies not showing any between-group differences, whereas the metabolites were significantly increased in patients with moderate and severe TBI.^44^ Creatinine values were not affected by age, sex or severity of injury.^44^

In conclusion, NAA increase is a pathophysiologically plausible phenomenon that appears to be linked to axonal dysfunction caused by mTBI. Its use as a prognostic marker has not yet been fully explored: a small study showed that NAA reduction in white matter is present only in mTBI patients that complained of post-concussive symptoms, suggesting that this metabolite might be a useful biomarker, but further validation, ideally in the context of multi-modal predictive models (e.g. incorporating other imaging sequences and/or other laboratory biomarkers), is necessary.^80^

### Susceptibility-weighted magnetic resonance imaging

It is possible to detect small bleeds using SWI sequences that are particularly sensitive in discriminating haemorrhages from venules even when lesions are smaller than 1 cm.^81^

Significantly more microhaemorrhages have been detected in mTBI compared with controls weeks and years post-injury.^82-84^ There is, however, a large retrospective assessment of 146 veterans who had sustained injury on average 9 years prior, which did not identify any SWI lesions in patients.^85^ A longitudinal assessment of 194 civilians showed that although number of lesions in an individual subject might not vary with time, they might become smaller and more inhomogeneous,^86^ offering a possible explanation for the reason why these lesions might be more difficult to detect many years post-injury.

Microbleeds can also be detected in healthy participants, but their anatomical distribution appears to be different in mTBI compared with healthy controls. In mTBI, SWI lesions are typically located cortically (often defined as contusions) and sub-cortically (often in conjunction with T_2_ hyperintensities and in the context of traumatic axonal injury), whereas they are mainly found in deeper white matter in controls.^82^ Frontal, parietal and temporal lobes as well as corpus callosum are mostly affected.^86-88^

On a group level, the proportion of patients with microbleeds detected by SWI increases with severity of injury,^83,89,90^ and females might be more affected,^84^ although pathophysiological reasons for sex differences are not known. Importantly, correlations with post-concussive symptomatology have not been consistent. Patients with microhaemorrhages have been reported to have lower scores on tests assessing short-term verbal and working memory, but not on tests of sustained attention.^82^ Wang *et al.* demonstrated that civilians who had mTBI and were depressed had significantly more and larger SWI microbleeds compared with non-depressed mTBI patients.^87^ However, another study assessing 127 patients with post-concussive complaints lasting for over 3 months did not find any associations between number of microhaemorrhages and symptoms.^88^ This lack of associations between symptoms and number of microhaemorrhages was replicated in 97 patients with persistent post-concussive headache compared with 96 health controls.^91^

Evidence to date suggests that future applications of SWI sequences could be in the diagnostic workup of mTBI, when presence of cortical contusions might corroborate a history of mTBI. At present, the potential of SWI as a sensitive prognostic biomarker appears to be quite limited.

### Fluid-attenuated inversion recovery

FLAIR sequences can be utilized to detect traumatic axonal injury: a well-recognized pathology underlying TBI, present in the majority of moderate and severe TBI, but only detectable in a minor proportion (∼7%) of mTBI patients.^90^

FLAIR sequences are also used to assess the burden of white matter hyperintensities, which present as punctuated lesions and have been reported to be the most common finding in TBI, detectable in over 50% of patients.^89^ Nonetheless, white matter hyperintensities are also well characterized in the general population on otherwise normal scans: they typically increase in number with ageing and might indicate an increased risk of cerebrovascular accidents and dementia.^92^

Although white matter hyperintensities are not unique to mTBI, they are more prevalent following head injury. A large study assessing 150 mTBI patients who had been referred for forensic examination identified sub-cortical and deep white matter hyperintensities in 42% of patients, a significantly larger proportion than in the 94 healthy controls (22%); the authors also noted that the difference was more pronounced in younger participants.^83^ Those findings were corroborated by another research comparing 138 young athletes with sport-related concussion to 136 non-concussed contact sportsmen and 96 non-concussed sportsmen controls.^84^ It is, however, important to note that although group-level changes have been demonstrated, white matter hyperintensities are not specific as they are present also in controls and might not be useful on an individual basis in the clinical settings. In addition, the reported proportion of hyperintensities in mTBI varies depending on studied cohorts, from 4^86^ to 42%.^83^

Similar to other structural markers above, some of the changes appear to improve over time. Specifically, a comprehensive longitudinal study scanning 194 mTBI patients at 72 h post-injury, at 3 months and after 1 year, reported that lesions attributed to traumatic axonal injury become less conspicuous and isointense over subsequent months, whilst punctuate lesions did not appear to change over time.^86^

In summary, white matter hyperintensities appear to be related to mTBI pathophysiology, but they are non-specific and appear not suitable, at least in isolation, as an imaging biomarker.

### Diffusion-weighted imaging

Diffusion-weighted imaging is the MRI technique of choice to evaluate the properties of white matter. Analogously to FLAIR, albeit with higher sensitivity, it is employed to identify traumatic axonal injury as well as more subtle alterations.^93^ Diffusion MRI parameters are related to direction and magnitude of water diffusion and usually based on diffusion tensor imaging (DTI) analysis, returning fractional anisotropy (FA), mean diffusivity (MD), axial diffusivity (Ad) and radial diffusivity (RD).

A recent systematic review appraised papers on diffusion MRI in mTBI published until May 2020. It highlighted that although there was substantial heterogeneity in methodology and results, there were some recurrent findings.^45^ Specifically, numerous studies conducted in acute and sub-acute mTBI detected increased MD and RD, with constant Ad and reduced FA in corpus callosum, internal capsule and corona radiata, a pattern indicative of diffuse axonal injury and associated vasogenic oedema, inflammation and, possibly, loss of myelin integrity.^45^ In fact, the pattern of reduced FA and increased MD, Ad and RD has recently been associated with concentration of blood neurofilaments, an emerging fluid biomarker of axonal damage and degeneration.^94^ Lindsey *et al.* also revealed a reverse pattern (reduced MD and RD with increased FA) affecting primarily dorsal cingulum and superior longitudinal fascicle. This was identified in fewer studies and suggested underlying cytotoxic oedema and apoptosis.^45^ Importantly, longitudinal studies and those conducted in chronic mTBI showed an incomplete normalization of diffusion metrics over time.^45^

The reported patterns of reduction in FA have since been replicated in large cohorts. Palacios *et al.* compared 391 patients and 148 controls corroborating findings of reduced FA and elevated MD, RD and Ad in corpus callosum and in projection and association fibres 2 weeks after injury. The mean deviation and Ad improved at 6 months, but other parameters did not normalize.^95^ Reduced FA in all lobes at white matter–grey matter interface was also shown in another large cohort (147 patients compared with 131 controls) 7.6 years post-injury,^65^ although a third large study did not detect any significant between-group differences in FA and MD.^96^

In recent years, associations between FA and clinical measures have emerged. Specifically, there is an inverse correlation between FA at white matter–grey matter junction and post-concussive symptomatology including slower processing and disruption in executive function.^65^ A significant association between FA in internal capsule and cerebral peduncles and anxiety and depression has also been reported^68^ as well as a link between reduced FA in splenium of corpus callosum and slower improvement of depressive symptomatology.^97^ Interestingly, the same study reported that elevated FA in genu and body of corpus callosum was predictive of faster resolution of cognitive symptoms.^97^

Ad has also been the focus of large research studies in past few years. For example, Vakhtin *et al.*^67^ assessed Ad in uncinate fascicle 18 years post-injury and found it to be significantly elevated in 171 veterans who had sustained mTBI compared with 115 veteran controls. Cai *et al.* suggested that global Ad is a predictor of emotional resistance in mTBI: this parameter was reduced in mTBI patients that had features of neuropsychiatric distress at 2 weeks post-injury compared with patients who had features of emotional resilience. In addition, Ad declined further at 6 months, but only in the neuropsychiatric distress group. Interestingly, association and projection fibres as well as fornix and superior cerebellar peduncle were the anatomical areas that contributed the most to significant changes.^98^

A novel diffusion technique, known as high-angular-resolution diffusion-weighted imaging and fixel analysis, has recently been employed in mTBI to estimate differences in fibre orientations within individual voxels (a feature that DTI is unable to provide). There is only one large study published to date showing no significant differences between 133 mTBI patients and 107 controls, although it is possible that results might have been affected by groups not having been matched for age, sex and education.^99^

In addition to DTI, there are novel diffusion MRI models that have demonstrated some success in identifying subtle changes after mTBI. For instance, diffusion kurtosis imaging (DKI) assumes that water diffusion can be also non-Gaussian and has been suggested to be more sensitive in the detection of microstructural changes associated with ageing.^100^ A study on 96 athletes with mTBI showed no significant differences in DTI metrics, but significantly higher axial kurtosis in corpus callosum 48 h post-injury. These changes were maintained after a week when they were detected also in corticospinal tract and superior and inferior longitudinal fascicles. In addition, at 2 weeks post-injury, radial kurtosis was found to be reduced, whereas kurtosis FA was increased in frontal lobe.^101^ Another study assessing both DTI and DKI parameters detected significantly lower FA and higher MD, Ad and RD in mTBI patients with persistent post-concussive symptomatology compared with controls, with differences reported in corpus callosum, corona radiata, internal capsule, superior longitudinal fascicle and thalamic radiation.^102^ The authors also argued for kurtosis analysis to be more sensitive as changes between mTBI patients without persistent post-concussion symptomatology and controls were noted only in kurtosis parameters.^102^ The same group assessed DTI and kurtosis longitudinally in 193 patients with mTBI and 83 controls, showing again significant between-group reductions in FA and mean kurtosis in all fibre types at baseline; however, longitudinal changes were equivocal, as they were driven by longitudinal variations in the control group.^103^ Diffusion kurtosis has also been used to explore pathophysiology: athletes who had suffered mTBI and who were also positive to cytomegalovirus had significantly higher axial and radial kurtosis and reduced mean cortical thickness compared with seronegative patients, suggesting that cytomegalovirus might predispose to tissue damage following mTBI.^104^

Taken together, DTI and DKI appear to be more sensitive in detecting white matter changes caused by mTBI compared with other MRI sequences. The results, nonetheless, are heterogeneous, and there are drawbacks related to requirements for complex analyses that might not be readily implementable in the clinical settings. Nonetheless, diffusion MRI is perhaps one of the techniques that has, so far, shown the most potential as a biomarker of tissue damage in mTBI.

### Functional magnetic resonance imaging

Functional magnetic resonance imaging is a widely used MRI technique that, through the blood-oxygen-level-dependent (BOLD) signal, measures regional variations in cerebral oxygenation, blood flow and blood volume secondary to local changes in neuronal activity.^105^

There are no large event-related fMRI studies published; hence, this section focusses on large studies on resting-state fMRI. Some resting-state fMRI studies have shown a generalized increase in functional connectivity in patients. This is typically interpreted as a compensatory mechanism to maintain function at pre-injury levels. For example, concussed athletes had higher local connectivity in the middle and superior frontal gyri 1 day post-injury which returned to normal levels when re-scanned in the asymptomatic stages. However, it is worth noting that elevated local connectivity at 24-h visit was associated with a subsequent increase in psychological symptoms.^106^ Another study detected increased functional connectivity in acute mTBI patients compared with healthy controls between inferior fronto-occipital fasciculus and primary sensorimotor cortex, normalizing in the chronic stages.^107^ Increased functional connectivity between periaqueductal grey matter, nucleus accumbens and rostral anterior cingulate cortex has also been reported,^108^ as well as hyperconnectivity of thalamus associated with emotional and cognitive symptoms.^109^ Li *et al.* focussed on network-level topological properties and showed enhanced functional connectivity in rich-club nodes and peripheral regions in acute mTBI.^110^ The same group also showed that dynamic cross-network interactions were significantly increased and were more variable in mTBI compared with controls. Specifically, mTBI had increased integration between the salience network and central executive network, but reduced coupling of the salience network with default-mode network. This increased network interaction was indicative of more severe cognitive impairments.^111^ Consistently, a deep learning approach has recently detected alterations in functional connectivity of default-mode and salience networks in mTBI, with high accuracy^112^ with a further study revealing alterations of functional decoupling of the default-mode network in chronic mTBI that was predictive of better cognitive outcomes.^113^

Interestingly, fMRI markers appear to be sensitive to the mode of injury with a few large studies revealing features that appear to be unique to blast mTBI. Specifically, blast mTBI patients have significant hyperconnectivity compared with controls and non-blast mTBI. Perhaps surprisingly, non-blast mTBI group showed significant hypo-connectivity compared with controls in a study comparing 186 blast patients to 80 non-blast patients with mTBI.^114^ Robinson *et al.*^115^ showed that close-range blast mTBIs are associated with altered functional connectivity in the primary somatosensory cortex and the pre-supplementary motor area compared with blasts at further distance. Gilmore *et al.* discovered an association between severity of blast mTBI and reduced functional connectivity in lateral geniculate body, medial frontal gyrus, lingual gyrus, right ventral anterior nucleus of thalamus and precuneus. The reduction in functional connectivity between visual and frontal areas was a predictor of worse outcome on WAIS digit-symbol coding task.^116^

In terms of the relationship between resting-state fMRI markers and symptoms, mTBI patients with PTDS appear to have increased connectivity patterns in parahippocampus and left middle frontal gyrus.^117^ In addition, there appears to be network decoupling related to re-experiencing symptoms, with severity of re-experiencing symptomatology being associated with fewer network connections.^118^

Functional MRI studies are unravelling complex network alterations following mTBI. They are extremely useful to explore pathophysiology and might, in the future, be of aid in assessing neuroimaging correlates of symptoms. However, due to complex analysis and heterogeneity of paradigms, fMRI is not yet suitable to be utilized as a biomarker in clinical practice.

### Arterial spin labelling

Arterial spin labelling is a functional MRI technique used to assess cerebral perfusion through quantification of cerebral blood flow. In ASL, water protons found in arterial blood are labelled magnetically, without the need to inject contrast.^119^

Analogously to event-related fMRI, there is a dearth of recent large studies assessing ASL in mTBI, with only one published in 2021, illustrating that number of injuries might be an important determinant of cortical perfusion. The authors showed correlations between number of blast exposures and increased perfusion in temporal cortex, anterior cingulate cortex and insula.^120^

However, a recent systematic review evaluated 23 studies using ASL in mTBI^46^ and concluded that it is likely that cerebral blood flow is reduced in the acute^121,122^ and sub-acute^122,123^ stages in frontal, parietal and occipital lobes, with some chronic changes reported in thalamus^124^ and temporal lobes of male athletes.^125^ Notably, some studies did not detect between-group differences in the first week following injury,^126-128^ whereas a few reported elevated cerebral blood flow in patients weeks to months post-concussion.^129,130^ Nonetheless, there is some evidence in support of the theory that a delayed cerebrovascular reaction may be taking place: cerebral blood flow has been shown to decrease over the weeks following injury in most longitudinal studies, with some recovery detected after 1 month.^131,132^ Sex could be a modifier of ASL parameters in mTBI: male patients demonstrated decreased perfusion compared with healthy male controls, whereas there was no between-group difference in females with and without mTBI.^125^ Notably, to date, there is still no consensus with regard to how post-concussive symptoms correlate with ASL measures, although larger studies in the field are missing.

Arterial spin labelling use in mTBI is still in the early stages; it is possible that it will emerge as a tool to prognosticate recovery, pending further validation studies in larger cohorts.

## Discussion

This review illustrates the numerous MRI modalities that have been utilized in mTBI research. Key highlights are illustrated in Figs 2–4. Table 1 summarizes the current state of the art detailed in this manuscript. Many MRI studies have been useful to elucidate pathophysiology although the potential of MRI as a biomarker in mTBI remains debated. To date, no MRI technique is ready to be utilized in isolation in clinical practice as a reliable diagnostic test or to predict outcomes.

**Table 1:** Key highlights of MRI sequences that have been utilized in mTBI research, including the settings in which they could be used, and possible future applications

MRI sequence	Settings	Current state of the art and required next steps
**T_1_-weighted magnetic resonance imaging**	Core sequence easily implementable in clinical scanners. Costs in line with those of clinical scans.	Useful in the assessment of brain aging following mTBI, possibly as a risk factor for neurodegenerative diseases. Unlikely to be useful in isolation as a biomarker of mTBI. There is no current evidence for significant volumetric alteration, but current studies are underpowered and, thus, large longitudinal studies in acute and sub-acute phases are required.
**Magnetic resonance spectroscopy**	Sequence used primarily in emerging research. It can be implemented in clinical scanners and in the clinical settings although local expertise and standardization of protocols are required which could increase costs.	Shows reduced N-acetyl-aspartate in the acute phases which is a non-specific finding, but indicative of cellular dysfunction. Sequence could be used in conjunction with other MRI sequences to characterize degree of damage and possibly help with prognostication in the context of a multi-parameter scoring system.
**Susceptibility-weighted magnetic resonance imaging**	Core sequence easily implementable in clinical scanners. Costs in line with those of clinical scans.	Able to detect small bleeds, cortical and sub-cortical contusions. Potentially useful in the diagnostic workup of patients, in the appropriate clinical context (e.g. patients with history of mTBI).
**Fluid-attenuated inversion recovery**	Core sequence easily implementable in clinical scanners. Costs in line with those of clinical scans.	Useful when traumatic axonal injury is detected (although this is in a minority of patients). In conjunction with other MRI markers, white matter hyperintensities burden might be useful to assess risk of developing long-term sequelae in the context of multi-parameter scoring system.
**Diffusion-weighted imaging**	High priority clinical research implementable in clinical scanners. It requires specific expertise regarding sequences and analysis, increasing costs.	Sensitive to changes in white matter structure that are related to pathophysiology. Need for standardization of protocols across sites and use of specific diffusion parameters (e.g. FA and Ad) in key anatomical areas (e.g. corpus callosum). It could be used in conjunction with other MRI techniques to compute the risk of developing long-term sequelae.
**Functional magnetic resonance imaging**	Emerging research implementable in clinical scanners. It requires specific expertise regarding sequences and analysis, increasing costs.	Sensitive in detecting mTBI-related changes to connectivity. Possible use to assess presence and resolution of specific post-concussive symptomatology (e.g. post-traumatic headache, cognitive dysfunction, emotional changes). Analysis standardization required.
**Arterial spin labelling**	High-utility emerging research implementable in clinical scanners. It requires specific expertise regarding sequences and analysis, increasing costs.	Initial results show alteration in cerebral blood flow in the acute and acute phases post-mTBI. It has potential as a biomarker of functional recovery, and it could be used in a multi-parameter scoring system to predict development of long-term sequelae and/or recovery. More studies in larger cohorts are needed.

MRI, magnetic resonance imaging; mTBI, mild traumatic brain injury.

Computed tomography in the emergency setting may exclude sinister pathologies and severe injuries, but it is not apt to investigate subtle changes of an mTBI. It also lacks fidelity for prognostication, especially in those patients with uncomplicated mTBI (i.e. mTBI with a normal CT head).^133^

T_1_-weighted MRI sequences are available on all clinical scanners and could be easily implemented in the workup of mTBI. The current literature, however, indicates that this modality is not sufficiently sensitive on an individual level as a confirmatory or prognostic test. Volumetric analysis has been useful to support the notion that mTBI contributes to accelerated brain ageing. Nonetheless, no large study, to date, has shown significant between-group differences in volumetric parameters or significant predictive potential of T_1_-weighted metrics. It is important to note that all large studies looking at differences between groups have acquired MRI many years post-injury and cross-sectionally; hence, assessment of acute and sub-acute changes in larger cohorts remains a gap in the field. Indeed, there is some evidence from smaller longitudinal studies suggesting that kinetics of changes following injury is complex. For instance, there are reported increases in volumes and cortical thickness immediately post-injury^134,135^ interpreted as mild brain oedema and reactive inflammatory response. These early changes are, however, postulated to be transitory: volumetric reductions and cortical thinning have been reported, again in smaller studies, in the months following injury^61,135-139^ and may represent a process either of resolution of early oedema and/or of cellular loss.

There are also inherent challenges associated with assessment of focal volumetric changes in mTBI linked to significant heterogeneity of mode and site of injury, all factors likely having a differential impact on cerebral areas: severity of injury has been suggested to be proportional to degree of volumetric loss^59^ as well as extent of thalamic atrophy.^62^ In addition, mode of injury (i.e. blast compared to non-blast) could differentially affect cortical thickness.^140^ It is also possible that individual changes may be lost when averaging results for group-level comparisons, rendering impractical the characterization of the specific effect of an injury in an individual patient. Differences inherent to scanning across sites^141^ and in software^142^ may also be partially responsible for discrepancies in outcomes and variability of results.

The main result of magnetic resonance spectroscopy is diffuse NAA reduction in mTBI. This is a non-specific finding consistently reported in neurological conditions.^143-146^ The metabolite is primarily located in neurons and is indicative of both neuronal function and integrity; it has also been shown to decline in parallel with disease progression in neurodegenerative pathologies.^143,144,146^ In the context of mTBI, NAA reductions indicate that, on a molecular level, neuronal dysfunction is present even in regions which might otherwise appear normal. Correlations with symptomatology and large longitudinal studies are needed to investigate the potential of this technique in mTBI. In addition, there are other heteronuclear MRS techniques such as phosphorus-31 MRS, which have shown potential in other diseases.^147,148^ Phosphorus-31 MRS has been recently employed to assess energy metabolism in moderate and severe TBI and showed pH and bioenergetic alterations in patients,^149,150^ although studies in mTBI are currently lacking. This technique could therefore be utilized in the future to explore specific pathophysiological mechanisms (e.g. bioenergetic alteration) in mTBI.

Microbleeds detected on SWI may emerge as an aid to diagnosis of TBI. Although not present in all cases, there is some evidence that cortical lesions (i.e. contusions) are a consequence of injury and relatively specific to traumatic events, within the context of head injury in young adults. Therefore, future use of SWI is likely to be in the diagnosis of mTBI rather than for prognostication, as associations with clinical measures and outcomes have been inconsistent. Even though they are both likely vascular in origin, microbleeds detected by SWI do not co-localize with hyperintensities on FLAIR.^89^ This suggests that they might be the result of different pathophysiological processes: the former might represent an acute haemorrhage, whereas the latter a result of chronic small vessel damage affecting myelination and possibly accelerated by traumatic injury. Novel techniques, such as the maximum ambiguity distance for phase imaging, are being developed to detect microbleeds with even greater precision and, although in need for further validation, they might be useful in future mTBI research.^151^ In addition, there are other susceptibility-weighted-based techniques, such as magnetic field correlation, which have been used to estimate non-heme iron load in the brain.^152^ It has been hypothesized that non-heme iron might accumulate in mTBI and be one of the links between traumatic injuries and neurodegenerative diseases;^153^ hence, potentials of this MRI modality might need exploring further.

FLAIR sequences are routinely utilized in clinical practice. They have a place in the identification of traumatic axonal injury, which is, however, only present in a minority of mTBI cases. Otherwise, the most prevalent findings are white matter hyperintensities which are small areas of demyelination likely secondary to small vessel damage.^154^ These are, however, neither sensitive nor specific to mTBI. Evidence for an association between white matter hyperintensity burden and memory impairment has emerged from studies conducted in small cohorts: white matter hyperintensity burden has been associated with working memory impairment^155^ and slower speed of information processing.^156^ Analogous findings were replicated in a study showing a larger proportion of frontal hyperintensities in military personnel with mTBI^58^ and in a study comparing 46 veterans with mTBI to 22 military controls: no between-group differences were found, but FLAIR hyperintensities correlated with short- and long-term memory impairment in the mTBI group, after correcting for PTSD symptoms.^157^ Although it is not possible to infer causation from these studies, these findings are an important first step in establishing an imaging biomarker of memory impairment in mTBI; for this purpose, FLAIR will likely need to be used in combination with other modalities.

Damage to white matter has been postulated to be one of the main pathologies in mTBI; hence, diffusion techniques have had a prominent role in research. Commissural, association and projecting fibres are all thought to be particularly vulnerable to blunt traumas: animal studies have shown that axons are mechanically disrupted when subjected to a differential motion between grey matter and deeper brain regions.^158^ In line with this hypothesis, DTI has shown alterations in corpus callosum and other white matter fibres although there are some discrepancies in published literature. Analogously to volumetric MRI, it is possible that heterogeneity in mode and type of injury as well as in analysis methodologies may be responsible for diverse results. The most consistent finding is a reduction in FA (associated with elevated MD and RD), correlating with post-concussive symptoms. This can result from a number of cellular processes such as vasogenic oedema, inflammation, demyelination and axonal injury. Some studies have explored the pathophysiology underlying FA changes in mTBI by combining multiple MRI techniques. The hypothesis is that SWI microvascular damage and myelin damage (on FLAIR) could affect connectivity. Microbleeds have indeed been shown to be associated with restricted diffusion in normally appearing white matter,^159-161^ and FA correlates with white matter hyperintensities.^162^ These findings indicate that FA alterations may be caused by numerous mechanisms in mTBI. Pleiotropy of pathophysiology affecting FA could, therefore, contribute to discrepancies in longitudinal studies assessing evolution of diffusion parameters. For instance, parameters altered following oedema are likely to normalize over time, whereas changes due to demyelination and axonal injuries could be longer lasting. In addition, differences in utilized sequences, field strengths as well as in type of analysis used might also be responsible for variations in study results. DTI and DKI appear to be sensitive to detection of pathophysiological processes related to mTBI and hence have potential as an imaging biomarker. There is, however, a great need for standardization of protocols, with analyses remaining complex and not immediately implementable in clinical practice. It is possible that diffusion techniques will prove clinically useful when utilized in conjunction with other MRI sequences (e.g. ASL) and taking into consideration the individual's clinical context.

Functional MRI investigations have been useful in elucidating pathophysiology. Resting-state fMRI has shown hyperconnectivity between numerous areas as well as biologically plausible correlations with symptomatology. There are smaller event-related fMRI studies which have shown alterations in functional activity associated with tasks probing emotional and cognitive function,^163^ with evidence suggesting a larger suppression of the default-mode network during cognitive tasks.^164^ In addition, there appears to be a differential response to emotions associated with neutral, fearful and negative stimuli.^165,166^ Event-related fMRI studies have also shown hyper-activation during working memory tasks.^167-170^ Albeit in need for replication in larger cohorts, event-related fMRI suggests the possibility of a compensatory increase in top-down attention control and recruitment of additional cerebral areas to compensate for subtle trauma-induced dysfunctions. Interpretation of data needs to be cautious and always in the context of clinical correlates, as there are numerous factors, such as PTSD and psychiatric comorbidities, that might affect results. Recently, fMRI has been used as a biomarker of treatment response in a preliminary study assessing efficacy of blue light therapy for mTBI.^171^ Although data were preliminary, it is possible that the technique might be utilized to evidence target engagement in future clinical trials targeting specific symptoms of mTBI.

Arterial spin labelling has been employed in some mTBI studies, primarily to assess putative delayed changes secondary to cerebrovascular injury. Alterations are thought to be a result of altered permeability of the blood–brain barrier as well as impairment of cerebral autoregulation, vasospasms and perfusion mismatch, with evidence coming from pre-clinical studies in animal models.^172^ Studies employing cerebrovascular reactivity and other MRI techniques assessing perfusion, such as quantitative dynamic contrast-enhanced MRI and dynamic susceptibility contrast MRI, are starting to corroborate these hypotheses.^126,173,174^ Although the kinetics of cerebral blood flow variations over time has not yet been satisfactorily elucidated, there is evidence to suggest that ASL may be a suitable MRI technique to assess recovery of mTBI, which is a necessary step in establishing a prognostic biomarker and warrants further investigation. In addition, a related measure, cerebrovascular reactivity, may be a promising and desirable measure to be used in conjunction or instead of cerebral perfusion measures.^172^ It is important to note that cerebrovascular reactivity can be measured also using Doppler methods and therefore is not unique to MRI although the precise relationship between the MRI and Doppler measures is yet to be established.^175^ In addition, there are other vascular imaging modalities, such as near-infrared spectroscopy, which may be utilized to assess putative vascular dysfunction. These techniques have a greater temporal resolution and potential to be recorded in field settings whilst assessing tasks related to common and real-life behaviours (e.g. postural shifts).^176^ Nevertheless, their limited spatial resolution means that they cannot replace MRI, but rather be used in combination to improve understanding of the impact of mTBI on cerebral vasculature.

Challenges related to identification of reliable imaging biomarkers in mTBI are due to significant heterogeneity of both techniques and pathology. Specifically, even within the same technique, there is large variability across studies in terms of scanner manufacturers, field strengths, hardware, sequences, acquisition parameters and analysis software. These differences inevitably affect sensitivity. For instance, most studies are conducted at 3T, although next-generation 7T and Connectome scanners could significantly improve specificity and sensitivity for mTBI.^177^ Higher field strength would improve SWI contrast, increase the BOLD effect (which becomes more focal to capillaries) and improve spectral resolution of MRS. In addition, at 7T, T_1_ relaxation is prolonged, offering the possibility to have different contrasts in anatomical sequences. That said, 7T scanners are scarce and at present limited to highly specialized research facilities, and thus, biomarkers that can be reliable identified at 3T have a much greater potential to become adopted as part of routine clinical practice in the near future. In addition, information on test–retest repeatability and reproducibility is often lacking. Some have attempted to conduct reproducibility studies,^178^ but, ideally, process should be iterative, to improve on technical standards that can then be applied in clinical practice. Therefore, efforts towards larger multi-centre studies and harmonization across sites are still necessary.^95,179^

This review illustrates that heterogeneity is also an inherent characteristic of the mTBI population. Type of injury (e.g. blast versus blunt), anatomical location of trauma, timing and number of injuries, presence of comorbidities (e.g. PTSD and depression), use of medications/drugs, age and population studied (civilians, military, athletes, mixed cohorts) can all have a significant impact on results. The picture is complicated by discrepancies in study design and diversity of diagnostic criteria utilized. Lastly, most clinical correlates rely on self-reported symptoms and questionnaires, which are affected by recall bias in the context of a disease which often affects memory. Therefore, there is an unmet need for large prospective multi-centre longitudinal studies that phenotype patients in depth at baseline and assess evolution MRI parameters concurrently with symptoms development to account for the abovementioned confounders. Prognostic models on the basis of admission characteristics have been developed in moderate and severe TBI and are currently used to predict outcomes;^180^ hence, the ultimate aim would be to develop a decision tree for mTBI to enable stratification of patients into risk categories.

It is possible that, in the future, there will be increasing use of multi-featured analysis incorporating volumetric data, MRS, FLAIR, DTI, SWI, ASL and fMRI in diagnosis, stratification of risk of sequelae and prognostication as well as for investigation and characterization of individual post-concussive symptoms. For example, there have been numerous studies aimed at characterizing post-traumatic headache, one of the most disabling and prevalent symptoms following mTBI. It has been shown that functional connectivity in mTBI patients with post-traumatic headache is altered and correlates with symptom severity^181,182^ in a way that is different compared with patients with migraine;^183^ hence, measures could potentially be developed to identify patients at risk of developing this disabling sequela. Deep learning models and machine learning classifiers to distinguish post-traumatic headache from healthy controls or patients with migraine have since been designed.^184-186^ For instance, utilizing a machine learning algorithm, Chong *et al.*^187^ reported that the individual MRI measures contributing the most to correct classification to differentiate persistent post-traumatic headache from migraine were MD in the right anterior thalamic radiation and RD in the right superior longitudinal fasciculus. These studies show how MRI, in conjunction with machine learning, could assist with classification, prognostication and potentially tracking recovery of specific post-concussive symptoms.

In summary, MRI is useful to elucidate pathophysiology *in vivo* and has shown associations with symptomatology, at group levels. Whilst this indicates that symptoms of mTBI are reflective of brain pathology, we still do not have an isolated imaging biomarker to be used in clinic. Arterial spin labelling and DTI have shown perhaps the greatest potential as biomarkers as they are most sensitive to different aspects of pathophysiology. It is likely that a combination of MRI sequences, possibly in conjunction with other brain imaging techniques (e.g. magnetoencephalography, Doppler, near-infrared spectroscopy) or blood biomarkers will be needed to establish reliable biomarkers of mTBI.^188-195^ In addition to utilizing imaging, broader use of standardized automated software and machine learning might be beneficial to bring advanced neuroimaging into clinical practice.

It is therefore recommended that larger longitudinal studies, focussing on promising techniques such as ASL in combination with DTI and DKI, are conducted. These studies would benefit from a deep phenotyping of mTBI patients and from subsequent multi-featured analyses, leading to a decision tree or scoring system to stratify patients with mTBI in clinical settings.

## Supplementary Material

fcaf120_Supplementary_Data

## Data Availability

Data sharing is not applicable as no primary data were generated from this review.

## Appendix

### Members of the UK mTBI Predict Consortium

Alexandra J. Sinclair, Aliza Finch, Adam Hampshire, Alice Sitch, Ali Mazaheri, Andrew P. Bagshaw, Asha Strom, Alice Waitt, Andreas Yiangou, Alexander Bennett, Angus Hunter, Caroline Witton, Davinia Fernández-Espejo, Dan Ford, Duncan Wilson, Hamid Dehghani, Hyojin Park, Hannah S. Lyons, Helen Brunger, Henrietta Ellis, Iman Idrees, Ian Varley, Jessica Hubbard, Jun Cao, Jon Deeks, James L. Mitchell, Jan Novak, Jamie Pringle, John Terry, Jack Rogers, Jessikah Fildes, Karen Mullinger, Lisa J. Hill, Mark Thaller, Martin Wilson, Matilde Sassani, Matthew Brookes, Ned Jenkinson, Ole Jensen, Pete Hellyer, Sebastian Coleman, Raymond Reynolds, Richard Blanch, Katie Morris, Ryan Ottridge, Rachel Upthegrove, Pradeepa R. W. Arachchige, Sarah Berhane, Samuel J. E. Lucas, Sophie Prosser, Shreshth Dharm-Datta, Tara Ghafari, Waheeda Hawa, Yidian Gao.
