# Supplementary material for: Current and prospective roles of magnetic resonance imaging in mild traumatic brain injury
Source: Brain Commun. 2025 Mar 25;7(2):fcaf120. doi: 10.1093/braincomms/fcaf120 (PMC12001801; doi:10.1093/braincomms/fcaf120)
Supplement: fcaf120_Supplementary_Data [file fcaf120_supplementary_data.zip › Supplementary_Appendix_1_Search strategy.docx]

***Search strategy:***

The following searches were performed in PubMed: 1. “(T1 weighted) AND (mild traumatic brain injury)”, 2. “(volumetric) AND (mild traumatic brain injury)”, 3. “(magnetic resonance spectroscopy) AND (mild traumatic brain injury)”, 4. “(susceptibility MRI) AND (mild traumatic brain injury)”, 5. “(fluid attenuated inversion recovery) AND (mild traumatic brain injury)”, 6. (FLAIR) AND (mild traumatic brain injury), 7. (mild traumatic brain injury) OR (concussion) OR (mild brain trauma mild head injury) AND (DTI) OR (diffusion tractography) OR (diffusion tensor imaging) OR (diffusion magnetic resonance imaging) OR (diffusion MRI) OR (diffusion weighted imaging) OR (diffusion weighted MRI) OR (anisotropy) OR (apparent diffusion coefficient) OR (mean diffusivity)”, 8. “(functional MRI) AND (mild traumatic brain injury)”, 9. “(arterial spin labelling) AND (mild traumatic brain injury)”.
