# Supplementary material for: Current and prospective roles of magnetic resonance imaging in mild traumatic brain injury
Source: Brain Commun. 2025 Mar 25;7(2):fcaf120. doi: 10.1093/braincomms/fcaf120 (PMC12001801; doi:10.1093/braincomms/fcaf120)
Supplement: fcaf120_Supplementary_Data [file fcaf120_supplementary_data.zip › Supplementary_Table_1.docx]

| ***Study*** | ***mTBI participants and controls*** | ***Type of injury*** | ***Age range of inclusion criteria in years (y)***  ***(mean age of mTBI cohort, number of male:female participants in mTBI cohort)*** | ***Timing of MRI scan*** | ***MRI sequences; strength of magnet*** | ***Main significant results*** | ***Significant correlates with clinical measures*** | ***Possible bias and limitations*** |
| --- | --- | --- | --- | --- | --- | --- | --- | --- |
| ^1^ | Civilians  137 mTBI, not depressed  28 mTBI with depression | Motor vehicle accidents, fall, assault, sport | 18-65 y  (mTBI without depressive disorder: 45 y, 83:54)  (mTBI with depressive disorder: 40 y, 20:8) | 2 hours to 3 days post-injury  Depression assessed at 1 year | SWI  (visual determination of lesions by two neuroradiologists);  3T | Significantly more and larger microbleeds in depressed group (71.4%) than non-depressed (8.8%).  Lesions in frontal, parietal, temporal lobes. | Not available. | No healthy control group.  Cross sectional MRI acquisitions.  A proportion of data was not analysed: patients lost to follow up and no information available on them developing depression.  Motion artefacts.  Manual and semi-quantitative analysis. |
| ^2^ | Civilians  111 isolated mTBI  111 healthy volunteers | Not available | Not available  (37 y, 43:68) | 25 days post-injury | T1w  T2w  FLAIR  DWI (b=1000)  SWI (SWAN, T2*w, assessed by two radiologists);  3T | Significantly more microbleeds in mTBI (23.4%) compared to controls (10.8%).  In mTBI, microbleeds were mainly in cortical and sub-cortical white matter, whereas in controls they were located mainly centrally. | mTBI with microbleeds had significantly lower scores on test assessing verbal short-term and working memory, compared to those without microbleeds, although there were no significant differences in tests assessing sustained attention. | No information on type of injury.  Cross sectional, no pre-injury scans or information. |
| ^3^ | Civilians  90 uncomplicated (i.e. no abnormalities on CT) isolated mTBI  78 complicated (abnormalities on CT) isolated mTBI | Road traffic accident, falls, violence | 16-65 y  (39 y, 54:36) | 4 weeks post-injury | T1w (Freesurfer);  3T | No significant differences between groups in all analyses (global volumetric, cortical volumes, and cortical thickness).  Larger ventricles in complicated mTBI compared to controls, but results not remaining significant after adjusting for age, sex, education and global intracranial volume. | Not available. | Cross sectional data, no control (healthy or orthopaedic injured).  No information regarding post-concussive symptomatology (neither in demographics of population, nor in analysis).  No information on pre-morbid status (i.e. prev PTSD, adverse life events ecc). |
| ^4^ | Military  768 mTBI  52 moderate TBI  14 severe TBI  42 active-duty controls | 688 with single or multiple blasts, other type of injuries not specified | For all TBI: 18-60 y  (34 y, 792:42) | 3.8 years post-injury | T1w (with and without contrast)  T2w  GRE  SWI  FLAIR (with and without contrast);  3T | Microhaemorrhage in whole TBI cohort: 7.2%.  Microhaemorrhages in mTBI cohort: 3.5%.  Microhaemorrhages in moderate and severe TBI: 47%.  No colocalization between microhaemorrhages and T2 hyperintensities. | Significantly more diffuse axonal injury, contusion, encephalomalacia, micro-haemorrhages, T2 enhancement, and gliosis in moderate and severe TBI compared to mTBI. | Radiologists not blinded to analysis.  Controls not matched for age and sex. Most reported analyses pooling all TBI.  Cross-sectional design. |
| ^5^ | Military veterans  105 veterans with lifetime history of mTBI  55 veterans without lifetime history of mTBI | Military and non-military mTBI  Blast, falls, motor vehicle accidents, physical assault, sport related injury, hit by blunt object | Not available  (31 y, 98:7) | Not available | T1w (cortical thickness, FreeSurfer);  3T | Relationship between polygenic risk score for developing Alzheimer disease and cortical thickness is mediated by mTBI. Area significant following correction for multiple comparisons: right posterior cingulate cortex.  No effect of Apo ɛ on cortical thinning, and no significant interaction of mTBI in this specific model. | Reduced delayed episodic memory was associated with cortical thinning in areas associated with Alzheimer’s disease. This effect this being mediated by polygenic risk score and presence of mTBI. | Groups not matched for PTSD.  No age range of participants, no time since last injury specified.  Self-reported mTBI.  Only military, not representative of entire mTBI population.  Female cohort exiguous in number.  Cross sectional design. |
| ^6^ | Military  89 Military with mTBI and 3 with severe TBI  34 Military without mTBI  762 Healthy controls from a publicly available database | Blasts and non-blasts | 18-60 y  (30 y, 88:4) | 50 months post-injury | T1w (cortical thickness used for estimation of brain age)  DWI (64 directions, b=1000, as part of brain age estimation imaging processing pipeline);  3T | No significant differences in cortical thickness in military with and without mTBI.  Age predicted was higher in military with mTBI compared to their actual age.  Significantly increased predicted age in military with mTBI compared to those without mTBI. | No correlations between estimated age and length of post-traumatic amnesia, number of blasts, time since injury, severity of PTSD, depression, alcohol consumption. | Retrospective design.  Cross sectional.  Diagnosis based on self-report.  Only military, not representative of entire mTBI population.  No information on pre-morbid psychiatric status |
| ^7^ | Civilians  147 mTBI | Motor vehicle accidents, falls, violence | 16-65 y  (40 y, 92:55) | 37 days post-injury | T1w (FreeSurfer, volumetrics, surface area, and cortical thickness)  T2w  SWI;  3T | Not available | Clinical parameters were predictive of outcomes of mTBI at 12 months. However, MRI did not improve clinical model, nor (when used in isolation) had significant predictive power. | A good prospective cohort design which also had information on pre-injury factors from medical literature including anxiety and depression.  Potential risk of overfitting the model as mTBI sample small for this kind of predictive studies.  No control group.  Cross-sectional.  Pre-injury characteristics based on self-report.  mTBI assessed in emergency department might represent more severe end of the spectrum. |
| ^8^ | Patients referred for forensic examination  150 mTBI  22 moderate TBI  8 severe TBI  94 healthy controls | Motor vehicle accidents, blunt forces, falls, sport, blast, assault | 14-81 y  (43 y, 102:78) | 29 months post-injury | SWI  FLAIR;  1.5T and 3T | Significantly more white matter hyperintensities on FLAIR in mTBI (42%) compared to controls (22%), with differences more pronounced in younger participants.  Hyperintensities were primarily found subcortically and in deep white matter.  Significantly more microhaemorrhages in mTBI (17%) compared to controls (3.2%). Age not a factor.  For mTBI, 52% of patient had either FLAIR and/or SWI abnormality on scan. | Probability of abnormal scan increases with severity of injury. | Retrospective case control design.  Different scanners and magnet strength used.  Possible selection bias of controls (recruited from hospital staff and students) |
| ^9^ | Not available  127 mTBI with persistent post-concussive complaints for over 3 months visiting level 1 trauma centre | Not available | 16-76 y  (39 y, 74:53) | 24 weeks post-injury | T1w  T2w  FLAIR  SWI  GRE (T2*)  (neuroradiologist visual assessment: MRI normal or not; then another researcher counted and localised micro-haemorrhages);  1.5 T | 63 had MRI abnormalities  64 no MRI abnormalities  SWI is more sensitive than GRE in detecting micro-haemorrhages (almost double detection)  Lesions are found primarily in frontal and temporal lobes. | No correlation between number of haemorrhages and number of post-traumatic complaints.  On multivariate regression, functional outcome was associated with loss of consciousness and temporal cortical microhaemorrhages on SWI. | Retrospective study. Patients group not matched.  No healthy control group.  Mechanism of injury not specified. |
| ^10^ | Military veterans  146 mTBI patients | Single and multiple blast mTBI, no blunt traumatic head injury | 18-70 y  (33 y, 132:14) | Mean 9.4 years post-injury | SWI (assessed by two neuroradiologists);  3T | No cerebral micro-haemorrhages detected. | Not available. | Retrospective study.  Self-reporting of mTBI.  Large variance in timing of scan since injury. |
| ^11^ | Military – consortium study including 31 centres  269 mTBI  50 controls | Deployment and non-deployment (penetrating injuries were excluded) | 22-69 y  (39 y, 234:35) | Average 8.8 years following worse injury reported | T1w (FreeSurfer);  Multi-site | No significant differences across 12 regions of interests between mTBI and non-mTBI. Significantly reduced volume of third ventricle in mTBI, however p value would not survive correction for multiple comparisons.  For surface analysis: significant differences in left hemisphere only (superior temporal gyrus, insula, frontal lobe and precuneus) | Inverse relationship between total brain volume and age.  Positive association between total brain volume and height, weight and body mass index.  Significantly differences across MRI sites. | Descriptive reporting of preliminary data (not full dataset), not hypothesis driven. Cross sectional.  Controls not matched for total combat exposure. In correlations, p values not corrected for multiple comparisons. |
| ^12^ | Sportsmen  138 contact sport athletes with mTBI  135 non-concussed contact sport athletes  96 non-concussed non-contact athletes | Sport-related concussion | Not available  (20 y,  124:14) | 24 hours post-injury  48 hours post-injury  7 days post-injury  6 months post injury | T1w  FLAIR  T2* and GRE  (assessed by three neuroradiologists);  3T | Any MRI finding in mTBI: 30.4%  Most common MRI finding on mTBI was white matter hyperintensities (13.8%) (Vs 6.7% of contact sport controls, and 6.3% of non-contact sport controls).  Chronic microhaemorrhages found in 2.2% of mTBI (Vs 0.7% of contact sport controls, and 1% of non-contact sport controls).  Female more likely to have findings than males.  mTBI athletes more likely to have findings than contact sport controls.  No significant differences between contact sport and non-contact sport controls. | Presence of mTBI, female, higher socioeconomic status, numbers of years spent participating in sport and being in division one were associated with higher incidence of MRI findings. | Different scanners.  Possibility by neuroradiologists to infer type of sport of participants.  Small number of female participants in mTBI cohort. |
| ^13^ | Civilians  194 mTBI patients  at 72 hours post injury  165 mTBI at 3 months post-injury  152 mTBI at 12 months post injury  78 community controls at first time point  73 community controls at 3 months  64 community controls at 12 months | Fall, violence, sport, motor vehicle accidents/bicycle, blunt trauma, others | 16-60 years  (27 y, 124:70) | 72 hours post-injury  3 months post-injury  12 months post-injury | T2w  DWI  FLAIR  SWI  (reviewed by two radiologist; microbleeds defined as traumatic axonal injury if in lobar white matter, corpus callosum, brainstem, basal ganglia or thalamus; defined as contusion if in cortex);  3 T | SWI lesions consistent with traumatic axonal injury found in 6% of patients (vs 2.5% of controls) and persisted at 12 months albeit smaller and less uniformly hypointense.  SWI lesions consistent with contusions found in 7% of patients, and persisted at 12 months.  SWI lesions located in frontal, temporal lobes and corpus callosum.  FLAIR lesions found in 4% of patients and found in fewer patients at 12 months, at 12 months lesions were more isointense.  SWI detected more lesions than FLAIR.  Diffusion lesions noted in 2% of patients. | Trend towards association between temporal lobe lesions and duration of post-traumatic amnesia (p=0.058). | Prospective recruitment.  Controls might have had previous mTBI (although at lower rate compared to previous mTBI in patients).  Challenging to differentiate traumatic axonal lesions from non-traumatic white matter abnormalities. |
| ^14^ | Civilians  162 mTBI patients  142 moderate TBI  186 severe TBI | Road traffic accidents, falls, violence, others | 16-60 y  (29 y, 107:55) | 2 days post-injury | DWI  FLAIR  SWI and GRE  (assessment of traumatic axonal injury and brain contusions);  1T, 1.5 T and 3T | Lesions consistent with traumatic axonal injury found in 7% of mTBI, 69% of moderate TBI and 93% of severe TBI. | Association between bilateral lesions in brainstem and GCS as well as between total number and volume of traumatic axonal injury and Glasgow coma scale and post-traumatic amnesia (NB analyses including all TBI).  No associations between contusions and Glasgow coma scale.  Total volume of traumatic axonal injury by DWI and FLAIR are better model for Glasgow coma scale score than using traumatic axonal injury on FLAIR + DWI + SWI.  Traumatic axonal injury used in conjunction with age and CT findings: area under the curve was 0.83 on Receiver Operating Characteristic curve predicting prolonged post-traumatic amnesia over 28 days. | MRI performed later in more severe cases of TBI.  Different scanners and different MRI protocols.  Microhaemorrhages quantified both from GRE and SWI.  No validate scale used to assess post-traumatic amnesia. |
| ^15^ | Not available. 11 academic centres, 1 trauma centre  421 mTBI | Road traffic accidents, incidents, falls, violence,  assault, others | > 17 y  (39 y, 280:141) | 2 weeks post-injury | T1w (Freesurfer);  3T | Not available. | Volume of superior frontal cortex, rostral anterior and caudal anterior cingulate cortex were predictive of PTSD at three months after accounting for numerous factors (sex, intracranial volume, ethnicity and race, education history, previous psychiatric illnesses and TBI, cause of injury, and PTSD at 2 weeks.  Those regions were not predictive of PTSD at six months. | Analysis focussing exclusively on specific regions of limbic system and frontal and cingulate cortices. Multiple sites.  No control group.  Cross sectional. No pre-injury scan. PTSD related to worst life trauma, not necessarily the one associated with mTBI following which participants were recruited. Effect size was significant but small, so the authors queried whether useful clinically. |
| ^16^ | Civilians  109 mTBI | Falls | 18-79 y  (40 y, 63:46) | 1-week post-injury  6 months post-injury | T1w (Freesurfer)  SWI (micro-haemorrhages – rated by three experts)  DWI (64 directions, average right-left fractional anisotropy, unscented Kalman filter tractography and feature selection by principal component analysis);  Strength of magnet not stated | Not available. | Age and fractional anisotropy decrease in corpus callosum, inferior and middle longitudinal fasciculi, inferior-occipitofrontal fasciculi, and superficial frontal and temporal fasciculi were linearly associated.  Fractional anisotropy of the corpus callosum, superficial frontal fasciculi, and corticospinal tract decreased significantly at six months.  Age and cerebral microbleeds contribute to fractional anisotropy decrease in genu of corpus callosum at six months. | At feature selection (conducted through principal component analysis), only structures which had bilateral principal component weight magnitudes were retained.  No healthy control group, unable to determine if changes were related to mTBI or solely aging.  Patients not screened for other diseases that might cause fractional anisotropy reduction and are associated with aging. |
| ^17^ | Active-duty military and veterans (8 sites)  822 participants  of which 667 had TBI  201 mTBI scanned longitudinally at 1.6 years  3377 controls from public database to train brain age model | Deployment and non-deployment related, blast and non-blast | > 18 y  (40 y, 714:201) | Time since injury of baseline scans not specified  Longitudinal scans conducted at 1.6 year (on average) | T1w (statistical parametric mapping, brain age prediction);  3T | mTBI male patients with deployment associated mTBI had higher brain age by 0.95 y.  No longitudinal changes. | Increase brain age was reported in PTSD, depression and alcohol abuse. | Prospective Longitudinal Study. Some of the results interpretation is challenging as not all demographic information (e.g. n of controls used) available.  Global assessment (rather than individual brain regions).  Small number of female participants.  No information on pre-morbid status. |
| ^18^ | Civilians  133 isolated mTBI  3377 healthy controls from database as training sample | Ground level fall | 20-83 y  (43 y, 82:51) | Baseline scan at 7 days post-injury  Follow up longitudinal scan at 6 months | T1w (brain age estimation)  3T | Patients with mTBI had increased brain age compared to chronological age, this was more pronounced in older (>40 year) adults (increase by 15 years) compared to younger (< 40 year) ones (in whom brain age was, on average 2 years higher).  No significant longitudinal changes in brain age estimation. | Not available. | No pre-injury scan and not possible to account for factors which might be confounders such as comorbidities (e.g. pre-injury mild cognitive impairment, hypertension ecc), medications, socio-economic status or lifestyle.  Training cohort from database, but no healthy controls.  Only one mTBI as inclusion criterion, only fall from standing as inclusion criterion.  No information on which anatomical area contributed to accelerated aging.  Power not sufficient to determine whether significant differences between sex groups.  Other measures of aging not used to validate results. |
| ^19^ | Civilians  113 mTBI  3418 controls (from repositories) | Ground level fall causing direct trauma | 21-95 y  (43 y, 69:44) | 24 weeks post-injury | T1w (Freesurfer, brain age)  Not available | mTBI predicted brain age was 9.2 years higher than healthy controls with equivalent chronological age. Anatomical structures driving the difference were: superior and middle frontal gyri, middle temporal gyrus, posterior dorsal cingulate gyrus and orbital sulci. | In mTBI, regional volumes are dependent on chronological age (but not sex). | Multiple acquisition sequences for T1w data.  Cross sectional.  Controls not prospectively recruited specifically for this study.  Subgroup of mTBI (i.e. only ground level falls causing direct trauma). |
| ^20^ | Not available  97 post-traumatic headache following isolated mTBI at least 12 month prior  96 healthy controls | Not available | 18-65 y  (mean 36, 17:80) | At least 12 months post-injury | FLAIR  SWI  3T | No significant between group differences in number of cerebral microbleeds (3.1% of mTBI scans) or white matter hyperintensities. | No correlations with clinical measures of anxiety, depression, cognition, sleep and headache disability. | Cross sectional, no information on pre-morbid status. No information on type of mTBI. |

Supplementary Table 1. Studies assessing mild traumatic brain injury (mTBI) utilising T_1_-weighted (T1w), susceptibility weighted (SWI), and/or fluid attenuation inversion recovery sequences (FLAIR). Other abbreviations: diffusion-weighted imaging (DWI), gradient echo (GRE), post-traumatic stress disorder (PTSD), T_2_-weighted (T2w), T2 star-weighted (T2*w), T2 star-weighted angiography (SWAN).

1. Wang X, Wei X-E, Li M-H, et al. Microbleeds on susceptibility-weighted MRI in depressive and non-depressive patients after mild traumatic brain injury. *Neurological Sciences*. 2014/10/01 2014;35(10):1533-1539. doi:10.1007/s10072-014-1788-3

2. Huang YL, Kuo YS, Tseng YC, Chen DY, Chiu WT, Chen CJ. Susceptibility-weighted MRI in mild traumatic brain injury. *Neurology*. Feb 10 2015;84(6):580-5. doi:10.1212/wnl.0000000000001237

3. Hellstrøm T, Westlye LT, Server A, et al. Volumetric and morphometric MRI findings in patients with mild traumatic brain injury. *Brain Inj*. 2016;30(13-14):1683-1691. doi:10.1080/02699052.2016.1199905

4. Riedy G, Senseney JS, Liu W, et al. Findings from Structural MR Imaging in Military Traumatic Brain Injury. *Radiology*. Apr 2016;279(1):207-15. doi:10.1148/radiol.2015150438

5. Hayes JP, Logue MW, Sadeh N, et al. Mild traumatic brain injury is associated with reduced cortical thickness in those at risk for Alzheimer's disease. *Brain*. Mar 1 2017;140(3):813-825. doi:10.1093/brain/aww344

6. Savjani RR, Taylor BA, Acion L, Wilde EA, Jorge RE. Accelerated Changes in Cortical Thickness Measurements with Age in Military Service Members with Traumatic Brain Injury. *J Neurotrauma*. Nov 15 2017;34(22):3107-3116. doi:10.1089/neu.2017.5022

7. Hellstrøm T, Kaufmann T, Andelic N, et al. Predicting Outcome 12 Months after Mild Traumatic Brain Injury in Patients Admitted to a Neurosurgery Service. *Front Neurol*. 2017;8:125. doi:10.3389/fneur.2017.00125

8. Trifan G, Gattu R, Haacke EM, Kou Z, Benson RR. MR imaging findings in mild traumatic brain injury with persistent neurological impairment. *Magn Reson Imaging*. Apr 2017;37:243-251. doi:10.1016/j.mri.2016.12.009

9. de Haan S, de Groot JC, Jacobs B, van der Naalt J. The association between microhaemorrhages and post - traumatic functional outcome in the chronic phase after mild traumatic brain injury. *Neuroradiology*. 2017/10/01 2017;59(10):963-969. doi:10.1007/s00234-017-1898-8

10. Lotan E, Morley C, Newman J, et al. Prevalence of Cerebral Microhemorrhage following Chronic Blast-Related Mild Traumatic Brain Injury in Military Service Members Using Susceptibility-Weighted MRI. *AJNR American journal of neuroradiology*. Jul 2018;39(7):1222-1225. doi:10.3174/ajnr.A5688

11. Bigler ED, Abildskov TJ, Eggleston B, et al. Structural neuroimaging in mild traumatic brain injury: A chronic effects of neurotrauma consortium study. *Int J Methods Psychiatr Res*. Sep 2019;28(3):e1781. doi:10.1002/mpr.1781

12. Klein AP, Tetzlaff JE, Bonis JM, et al. Prevalence of Potentially Clinically Significant Magnetic Resonance Imaging Findings in Athletes with and without Sport-Related Concussion. *J Neurotrauma*. Jun 2019;36(11):1776-1785. doi:10.1089/neu.2018.6055

13. Einarsen CE, Moen KG, Håberg AK, et al. Patients with Mild Traumatic Brain Injury Recruited from Both Hospital and Primary Care Settings: A Controlled Longitudinal Magnetic Resonance Imaging Study. *J Neurotrauma*. Nov 15 2019;36(22):3172-3182. doi:10.1089/neu.2018.6360

14. Moe HK, Follestad T, Andelic N, et al. Traumatic axonal injury on clinical MRI: association with the Glasgow Coma Scale score at scene of injury or at admission and prolonged posttraumatic amnesia. *J Neurosurg*. Oct 23 2020:1-12. doi:10.3171/2020.6.jns20112

15. Stein MB, Yuh E, Jain S, et al. Smaller Regional Brain Volumes Predict Posttraumatic Stress Disorder at 3 Months After Mild Traumatic Brain Injury. *Biol Psychiatry Cogn Neurosci Neuroimaging*. Mar 2021;6(3):352-359. doi:10.1016/j.bpsc.2020.10.008

16. Robles DJ, Dharani A, Rostowsky KA, et al. Older age, male sex, and cerebral microbleeds predict white matter loss after traumatic brain injury. *Geroscience*. Oct 26 2021;doi:10.1007/s11357-021-00459-2

17. Dennis EL, Taylor BA, Newsome MR, et al. Advanced brain age in deployment-related traumatic brain injury: A LIMBIC-CENC neuroimaging study. *Brain Inj*. Apr 16 2022;36(5):662-672. doi:10.1080/02699052.2022.2033844

18. Amgalan A, Maher AS, Ghosh S, Chui HC, Bogdan P, Irimia A. Brain age estimation reveals older adults' accelerated senescence after traumatic brain injury. *Geroscience*. Oct 2022;44(5):2509-2525. doi:10.1007/s11357-022-00597-1

19. Shida AF, Massett RJ, Imms P, Vegesna RV, Amgalan A, Irimia A. Significant Acceleration of Regional Brain Aging and Atrophy After Mild Traumatic Brain Injury. *J Gerontol A Biol Sci Med Sci*. Aug 2 2023;78(8):1328-1338. doi:10.1093/gerona/glad079

20. Ashina H, Christensen RH, Al-Khazali HM, et al. White matter hyperintensities and cerebral microbleeds in persistent post-traumatic headache attributed to mild traumatic brain injury: a magnetic resonance imaging study. *The Journal of Headache and Pain*. 2023/02/24 2023;24(1):15. doi:10.1186/s10194-023-01545-w
