# Supplementary material for: Current and prospective roles of magnetic resonance imaging in mild traumatic brain injury
Source: Brain Commun. 2025 Mar 25;7(2):fcaf120. doi: 10.1093/braincomms/fcaf120 (PMC12001801; doi:10.1093/braincomms/fcaf120)
Supplement: fcaf120_Supplementary_Data [file fcaf120_supplementary_data.zip › Supplementary_Table_2.docx]

| ***Study*** | ***mTBI participants and controls*** | ***Type of injury*** | ***Age range of inclusion criteria in years (y)***  ***(mean age of mTBI cohort, number of male:female participants in mTBI cohort)*** | ***Timing of MRI scan*** | ***MRI sequences; strength of magnet*** | ***Main significant results*** | ***Significant correlates with clinical measures*** | ***Possible bias and limitations*** |
| --- | --- | --- | --- | --- | --- | --- | --- | --- |
| ^1^ | Civilians  134 mTBI  15 moderate TBI  16 severe TBI  47 orthopaedic controls  59 healthy controls (family or friends) | Non-penetrating injury: motor vehicle accidents, bicycle accidents, falls, assaults, sport injuries | 19-80 y  (44 y, 104:30) | 6.7 months post-injury | DTI (64 direction, b=3000, region of interest analysis, FMRIB’s Diffusion Toolbox);  3T | No significant differences in fractional anisotropy or mean diffusivity between mTBI and all controls. | No significant differences in cognitive tests between mTBI and all controls.  Relationships between fractional anisotropy and mean diffusivity in mTBI and cognitive scores were not assessed. | Groups (TBI compared to all controls) not matched for age, sex, and education. No matching between timing of injury in orthopaedic controls and TBI.  Cross sectional. |
| ^2^ | Civilians  133 mTBI  15 moderate TBI  14 severe TBI  107 healthy and orthopaedic controls | Motor vehicle accidents, falls, bicycle, assaults, sports, pedestrian, others | 18-80 y  (44 y, 103:30) | 7 months post-injury | Diffusion (64 direction, b=3000, fixel based analysis);  3T | No significant differences between mTBI and controls in any assessed parameters. | Moderate-severe TBI had significantly lower fibre density, fibre-bundle cross section, and combined fibre density with fibre-bundle cross section in corpus callosum, internal and external capsule and cerebral peduncles in moderate-severe TBI compared to controls. | Group not matched for age, sex, and education.  Cross sectional. |
| ^3^ | Athletes  96 mTBI  82 controls | Sport related | Not available  (18 y, 96:0) | 48 hours post-injury  8 days post-injury  15 days post-injury  45 days post-injury | DTI and DKI (30 diffusion directions, b=1000 s/mm^2^ and b=2000 s/mm^2^, diffusional kurtosis estimator software, tract-based spatial statistics, whole brain voxel-based analysis and region of interest analysis);  3T | No between-group and longitudinal differences in DTI parameters (fractional anisotropy, mean diffusivity, radial diffusivity, axial diffusivity).  Axial kurtosis significantly elevated in patients compared to controls (but no significant changes in voxel-based analysis). At baseline scan, significant changes were localised to the corpus callosum. After a week, aside from corpus callosum, the corticospinal tract superior and inferior longitudinal fascicles also showed significant changes.  No significant between-group changes were found at 45 days.  Radial kurtosis significantly reduced in patients in frontal lobe at two weeks.  Kurtosis fractional anisotropy increased in patients at 15 at 45 days in frontal lobe. | No associations with clinical measures (duration and severity of symptoms). | No female participants, no age range specified. Controls had previous concussions.  Relatively low-resolution scan. |
| ^4^ | Civilians  123 mTBI  48 apolipoprotein E ɛ4 carriers with mTBI (18 female)  75 apolipoprotein E ɛ4 non-carriers with mTBI (30 female) | Traffic accidents, falls, violence, others | 16-65 y  (39 y, 75:48) | 12 months post-injury | T1w (estimation of brain age)  DTI (30 directions, b=1000, tract-based spatial statistics);  3T | Following false discovery rate correction, no significant differences in volumetric (subcortical and cortical volumes and cortical thickness), DTI, and brain-age gap in ApoE ɛ4 carriers compared to non-carriers. | Not available. | Cross sectional, with scans at 12 months. Relatively small cohort of Apo ɛ4 positive patients.  No controls without mTBI/no head injury. |
| ^5^ | Civilians  176 mTBI  78 community controls | Falls, bicycle, violence, sports, motor vehicle accidents, hit by an object, other/unknown | 16-59 y  (median=28 y, 111:65) | 72 hours post-injury | DTI and DKI (30 directions, b=1000, b=2000, tract-based spatial statistics);  3T | Significant between group differences between mTBI without persistent post-concussive symptoms and healthy controls only in kurtosis parameters in internal capsule, cerebellum, brainstem, and thalamus.  Patients with mTBI and persistent (>3 months) post-concussive symptoms had reduced fractional anisotropy, kurtosis fractional anisotropy, mean kurtosis, axial kurtosis and radial kurtosis compared to controls. They also had higher mean diffusivity, axial diffusivity and radial diffusivity. Most of the effect size was from corpus callosum with some differences in mean kurtosis reported in cerebellum and brainstem. | Reduced fractional anisotropy and kurtosis fractional anisotropy, elevated radial diffusivity in those with persistent (>3 months) post-concussive symptoms.  Most of the effect from corpus callosum, corona radiata, internal capsule, thalamic radiation. | Cross sectional design.  Scanner upgrade took place during study, this was a factor controlled and accounted for in analyses.  Multiple comparison correction carried out at voxel level, but not for numerous comparisons including different diffusion parameters and clinical subgroups. |
| ^6^ | Veterans  171 veterans with mTBI (sustained in their 20s)  115 veteran controls without mTBI history | Injury during military service | 23-77 y  (46 y, 158:13) | 18 years post-injury | T1w (Freesurfer)  DWI (b=1000, 60 directions, automated fibre quantification);  3T | Significant between group effect: increased axial diffusivity in uncinate fascicle in mTBI.  No significant between-group differences in whole brain or cortical grey matter volumes (although volumes did decrease with ageing). | Significant interaction (non-linear model) between white matter changes and age in radial diffusivity and mean diffusivity and axial diffusivity in anterior thalamic fascicles, corticospinal tract, inferior longitudinal fascicles, arcuate fascicles, uncinate fascicles. I.e. those changes were accelerated with increasing age in the mTBI group. No significant interaction between group and ageing on the effects of fractional anisotropy, whole brain white matter volume, cortical grey matter volumes. | Data acquired for clinical purposes. No reverse-phase encoding data to correct for putative susceptibility effects.  Cohort not matched for sex.  Only veterans.  Cross sectional design.  Unknown specifics of injury type.  No clinical correlations related to post-concussive symptomatology. |
| ^7^ | Not available  391 patients  148 healthy controls (friends or family of mTBI patients) | Traffic, fall, other, violence and assault | 17-60 y    (35 y, 269:132) | 2 weeks post-injury  6 months post-injury | DTI (64 diffusion encoding directions, b=1300, tract-based spatial statistics, whole brain);  3T | At 2 weeks, mTBI had significantly higher axial diffusivity, mean diffusivity and radial diffusivity than controls, largest effect size was found for axial diffusivity in body of corpus callosum and association fascicles (especially superior longitudinal fascicle, external capsule), and projection fibres (anterior limb of internal capsule and superior corona radiata).  At 2 weeks, fractional anisotropy was significantly reduced in association tracts (external capsule, superior fronto-occipital fascicle) and genu of corpus callosum.  Longitudinally, no changes in fractional anisotropy and radial diffusivity were detected, whereas axial diffusivity and mean diffusivity decreased significantly over time in the mTBI cohort. Axial diffusivity, mean diffusivity, and radial diffusivity were significantly elevated in mTBI at six months compared to controls, and fractional anisotropy was significantly reduced in mTBI compared to controls. | Axial diffusivity, and to a lesser extend mean diffusivity (but not fractional anisotropy or radial diffusivity) at 2 weeks were associated with complete recovery at six months post-injury.  Tract with most prognostic value were long association tracts in the left cerebral hemisphere: superior longitudinal fasicle, superior fronto-occipital fascicle, and external capsule. | Multicentre (11 centres, 13 scanners, Track-TBI) prospective. 20% of patients excluded due to motion artefacts.  Those over 60 years were excluded from the analysis. |
| ^8^ | Not available  172 mTBI  (94 emotional resilience compared to  78 neuropsychiatric distress)  148 uninjured controls | Road traffic accidents, falls, violence, assault, other | 17-60 y  (36 y, 118:54) | 2 weeks post-injury  6 months post-injury | DWI (DTI, b=1300, 64 directions, region of interest whole brain voxel-wise analysis);  3T | Global axial diffusivity reduced in neuropsychiatric distress compared to emotional resilience sub-groups at both time-points. Areas that contributed the most to changes were in association and projection fibres as well as fornix and superior cerebellar peduncle.  Global axial diffusivity remained stable in emotional resilience group but significantly decreased in neuropsychiatric distress group.  Uninjured controls had stable axial diffusivity over time. | Not available. | mTBI sub-groups were not matched for sex, education, race, cause of injury.  Multiple scanners (TrackTBI).  No correlations with clinical measures assessed.  Axial diffusivity only reported (not fractional anisotropy nor mean diffusivity). |
| ^9^ | Civilians, litigation  446 mTBI  with symptoms > 6 months | Fall, falling objects, mostly motor vehicle accidents, other, unknown | 13-82 y  (42 y, 184:262) | 100 days post-injury | DTI (at least 33 direction, Fiber Assignment with Continuous Tracking, region of interest analysis; fractional anisotropy in corpus  callosum);  3T | Higher fractional anisotropy in those < 40 years compared to those over 40.  No differences in fractional anisotropy between male and female.  No differences in fractional anisotropy between those with loss of consciousness and those without. | Higher fractional anisotropy in corpus callosum (genu and body) is prognostic for faster recovery from cognitive symptoms.  Lower fractional anisotropy in splenium of corpus callosum was associated with slower resolution of depression and emotional lability. | Retrospective study. Multi-site with multiple (eight) scanners and varying acquisition parameters.  No healthy control group. |
| ^10^ | Military and civilians  147 mTBI  131 controls | Not available | 18-70 y    (36 y, 96:51) | 7.6 years post-injury | T1w (whole brain cortical thickness, FreeSurfer)  DTI (tract-based spatial statistics);  3T | Reduced fractional anisotropy at grey matter to white matter boundaries in all lobes and in deep white matter in mTBI.  No significant difference in cortical thickness between groups. | Significant inverse correlation between post-concussive symptomatology and fractional anisotropy at white matter-grey matter boundary in whole brain, frontal and parietal lobes.  Significant correlation between functional status and fractional anisotropy at grey matter to white matter boundaries in all lobes and in deep white matter.  Significant inverse correlation between slower processing speed and fractional anisotropy at white matter-grey matter boundary in whole brain, frontal and temporal lobes.  Significant inverse correlation between executive function and fractional anisotropy at white matter-grey matter boundary in parietal lobe. | Multiple (six) sites and scanners, different acquisition parameters.  No orthopaedic injured controls. Patients and controls not matched for age.  Only assessed fractional anisotropy.  Cross sectional.  Gender assessed (not biological sex). Self-report of mTBI.  No information on mode of injury. |
| ^11^ | Not available  93 mTBI (40 with positive CT findings, 53 with negative CT findings)  21 controls with orthopaedic injuries, did not receive MRI | Road traffic accidents, falls, violence and assault, suicide attempt, others | > 18 y  (46 y, 64:29) | 7.5 months post-injury | FLAIR  SWI  DWI (b=1000 s/mm^2^, 64 phase encoding directions, tract-based spatial statistics);  3T | Significantly lower fractional anisotropy, and higher mean diffusivity, axial diffusivity, radial diffusivity in patients with CT findings compared to those with negative CT. | Significant inverse correlation between blood neurofilaments and fractional anisotropy. Significant positive correlation between blood neurofilaments and mean diffusivity, axial diffusivity, radial diffusivity. | Controls did not have MRI. Cross sectional study. Most, but not all, blood samples were taken within 24 hours from admission. mTBI patient cohort had more severe disease compared to most other studies (as mTBI was classified only on basis of Glasgow coma scale). |
| ^12^ | Athletes  88 mTBI (30 cytomegalovirus positive)  73 un-injured athletes (21 cytomegalovirus positive) | Sport related | Not available  (19 y, 88:0) | 24 hours post-injury  8 days post-injury  15 days post-injury  45 days post-injury | T1w (FreeSurfer, mean cortical thickness, surface area)  DKI (b=1000 and b=2000, 30 phase encoding directions);  3T | Patients with cytomegalovirus had  significantly higher axial and radial kurtosis and reduced mean cortical thickness compared to seronegative patients.  There were no significant changes in volumetric and DKI parameters in controls with and without cytomegalovirus. | Not available. | No females in cohort. Cross sectional, subgroup of patients and controls with cytomegalovirus was relatively small. |
| ^13^ | Civilians  193 mTBI  (complicated TBI defined as visible MRI lesions n=22)  83 controls | Falls, bicycle, sport injury, motor vehicle accidents, hit by object, other, unknown | 16-59 y  (27 y, 123:70) | 72 hours post-injury  3 months post-injury  12 months post-injury | T1w  T2w  FLAIR  SWI  DWI (DTI/DKI 30 directions, b=1000 and b=2000 FSL tract-based spatial statistics);  3T | Fractional anisotropy and mean kurtosis were lower in mTBI compared to controls in projection, association and commissural tracts.  Significant changes between mTBI and controls were seen longitudinally, but these changes were driven by alterations in parameters of controls rather than mTBI.  11 traumatic axonal injury  12 contusions. | Not available. | No significant differences between complicated and uncomplicated mTBI.  Scanner upgrade took place (but accounted for in analysis).  Only voxels found to be significantly different at baseline werer analysed longitudinally. |
| ^14^ | Not available  89 mTBI | Not available | 16-80 y  (37 y, 60:29) | 2 weeks post-injury | T1w (FreeSurfer: cortical thickness and subcortical volumes)  DWI (b=1000s/mm^2^, 55 diffusion encoding directions, DTI FSL BEDPOSTX, perivascular spaces);  3T | Not available. | Significant correlations between:  - Anxiety and cortical thinning (inferior operculum, occipital lobe and putamen); fractional anisotropy in superior longitudinal capsule, internal and external capsule, and cerebral peduncle.  - Depression and reduced thickness in areas in all lobes, cingulate gyrus, insula, and operculum; fractional anisotropy in cerebral peduncle and internal capsule.  - Somatisation and perivascular spaces; thalamic, amygdala and hippocampus, and white matter volumes; reduced mean diffusivity and widespread white matter tracts.  - Life satisfaction showed no significant correlations with imaging. | Multicentre (18 centres). Cross sectional. No control group. |

Supplementary Table 2. Studies assessing mild traumatic brain injury (mTBI) utilising diffusion weighted imaging (DWI) and sequences including diffusion tensor imaging (DTI) and diffusion kurtosis imaging (DKI). Other abbreviations: computed tomography (CT), fluid attenuation inversion recovery sequences (FLAIR), FMRIB Software Library (FSL), susceptibility weighted imaging (SWI), T_1_-weighted (T1w), T_2_-weighted (T2w).

1. Wallace EJ, Mathias JL, Ward L, Pannek K, Fripp J, Rose S. Chronic white matter changes detected using diffusion tensor imaging following adult traumatic brain injury and their relationship to cognition. *Neuropsychology*. Nov 2020;34(8):881-893. doi:10.1037/neu0000690

2. Wallace EJ, Mathias JL, Ward L, Fripp J, Rose S, Pannek K. A fixel-based analysis of micro- and macro-structural changes to white matter following adult traumatic brain injury. *Hum Brain Mapp*. Jun 1 2020;41(8):2187-2197. doi:10.1002/hbm.24939

3. Muftuler LT, Meier TB, Keith M, Budde MD, Huber DL, McCrea MA. Serial Diffusion Kurtosis Magnetic Resonance Imaging Study during Acute, Subacute, and Recovery Periods after Sport-Related Concussion. *J Neurotrauma*. Oct 1 2020;37(19):2081-2092. doi:10.1089/neu.2020.6993

4. Hellstrøm T, Andelic N, de Lange AG, Helseth E, Eiklid K, Westlye LT. Apolipoprotein ɛ4 Status and Brain Structure 12 Months after Mild Traumatic Injury: Brain Age Prediction Using Brain Morphometry and Diffusion Tensor Imaging. *J Clin Med*. Jan 22 2021;10(3)doi:10.3390/jcm10030418

5. Stenberg J, Eikenes L, Moen KG, Vik A, Håberg AK, Skandsen T. Acute Diffusion Tensor and Kurtosis Imaging and Outcome following Mild Traumatic Brain Injury. *J Neurotrauma*. Sep 15 2021;38(18):2560-2571. doi:10.1089/neu.2021.0074

6. Vakhtin AA, Zhang Y, Wintermark M, Ashford JW, Furst AJ. Distant histories of mild traumatic brain injury exacerbate age-related differences in white matter properties. *Neurobiol Aging*. Nov 2021;107:30-41. doi:10.1016/j.neurobiolaging.2021.07.002

7. Palacios EM, Yuh EL, Mac Donald CL, et al. Diffusion Tensor Imaging Reveals Elevated Diffusivity of White Matter Microstructure that Is Independently Associated with Long-Term Outcome after Mild Traumatic Brain Injury: A TRACK-TBI Study. *J Neurotrauma*. Oct 2022;39(19-20):1318-1328. doi:10.1089/neu.2021.0408

8. Cai LT, Brett BL, Palacios EM, et al. Emotional Resilience Predicts Preserved White Matter Microstructure Following Mild Traumatic Brain Injury. *Biol Psychiatry Cogn Neurosci Neuroimaging*. Sep 21 2022;doi:10.1016/j.bpsc.2022.08.015

9. Asturias A, Knoblauch T, Rodriguez A, et al. Diffusion in the corpus callosum predicts persistence of clinical symptoms after mild traumatic brain injury, a multi-scanner study. *Front Neuroimaging*. 2023;2:1153115. doi:10.3389/fnimg.2023.1153115

10. Pankatz L, Rojczyk P, Seitz-Holland J, et al. Adverse Outcome Following Mild Traumatic Brain Injury Is Associated with Microstructure Alterations at the Gray and White Matter Boundary. *J Clin Med*. Aug 21 2023;12(16)doi:10.3390/jcm12165415

11. Hossain I, Mohammadian M, Maanpää HR, et al. Plasma neurofilament light admission levels and development of axonal pathology in mild traumatic brain injury. *BMC Neurol*. Aug 15 2023;23(1):304. doi:10.1186/s12883-023-03284-6

12. Savitz J, Goeckner BD, Ford BN, et al. The effects of cytomegalovirus on brain structure following sport-related concussion. *Brain*. Oct 3 2023;146(10):4262-4273. doi:10.1093/brain/awad126

13. Stenberg J, Skandsen T, Moen KG, Vik A, Eikenes L, Håberg AK. Diffusion Tensor and Kurtosis Imaging Findings the First Year following Mild Traumatic Brain Injury. *J Neurotrauma*. Mar 2023;40(5-6):457-471. doi:10.1089/neu.2022.0206

14. Sibilia F, Custer RM, Irimia A, et al. Life After Mild Traumatic Brain Injury: Widespread Structural Brain Changes Associated With Psychological Distress Revealed With Multimodal Magnetic Resonance Imaging. *Biological Psychiatry Global Open Science*. 2023/07/01/ 2023;3(3):374-385. doi:<https://doi.org/10.1016/j.bpsgos.2022.03.004>
