# Supplementary material for: Current and prospective roles of magnetic resonance imaging in mild traumatic brain injury
Source: Brain Commun. 2025 Mar 25;7(2):fcaf120. doi: 10.1093/braincomms/fcaf120 (PMC12001801; doi:10.1093/braincomms/fcaf120)
Supplement: fcaf120_Supplementary_Data [file fcaf120_supplementary_data.zip › Supplementary_Table_3.docx]

| ***Study*** | ***mTBI participants and controls*** | ***Type of injury*** | ***Age range of inclusion criteria in years (y)***  ***(mean age of mTBI cohort, number of male:female participants in mTBI cohort)*** | ***Timing of MRI scan*** | ***MRI sequences; strength of magnet*** | ***Main significant results*** | ***Significant correlates with clinical measures*** | ***Possible bias and limitations*** |
| --- | --- | --- | --- | --- | --- | --- | --- | --- |
| ^1^ | Military veterans  41 close-range blast mTBI  98 no close-range blast mTBI | Blasts | 19-62 y  (33 y, 120:19) | 36 months post-deployment, and 5 years from most severe TBI | Resting-state fMRI  3T | Blast exposure at close range (<10 meters) was associated with altered functional connectivity in the default mode network and associated with decreased connectivity of the primary somatosensory cortex and the pre-supplementary motor area compared to those who had not experienced a blast at this proximity range.  No differences in functional connectivity in relation to past concussions or blast exposures at greater distances. | Connectivity differences due to close-range blast were not attributable to sleep quality, pain, length of/time since deployment, or PTSD symptoms. | No control group.  Assessments long removed from injury.  Retrospective and self-reported blast history.  Only military, not representative of entire mTBI population.  There were significant differences between close-range blast and no close-range blast in the following characteristics: number and duration of deployments, number of lifetime TBIs, military TBI and blast TBIs and PTSD which were all greater in close-range blast group. |
| ^2^ | Military veterans  208 subjects, of which  131 with mTBI | Blast | Not available  (32 y, 188:20) | Not available | Resting-state fMRI  3T | Hippocampal $\leftrightarrow$ prefrontal cortex decoupling evidenced at higher levels of re-experiencing.  Network decoupling related to re-experiencing was observed only in mTBI, indicating that mTBI may facilitate the link between re-experiencing symptoms and decoupling.  Re-experiencing severity predicted lower density: higher re-experiencing was associated with fewer network connections.  Worse local efficiency for right rostral middle frontal gyrus and posterior cingulate cortex suggesting higher re-experiencing is linked to greater inefficiency of communication in the networks surrounding these regions.  Decreased participation coefficient indicated that the right hippocampus interacts with fewer functional modules in high re-experiencing.  Re-experiencing linked to worse functional segregation (local efficiency). No differences in functional integration reported. | Relationship to functional disability:  Decreased right hippocampal $\leftrightarrow$right prefrontal cortex coupling associated with worse daily functioning on the World Health Organization Disability Assessment Schedule II with the mean connection correlating with the number of days those veterans reported reduced/were completely unable to carry out their usual activities.  Reduced right hippocampus degree was related to higher number of days veterans had to reduce their usual activities.  Interaction between mTBI and worse local efficiency in the right and left caudate predicted the number of days veterans reported having to reduce usual activities/being completely unable to carry out usual activities. | No control group.  Unclear demographics of sample assessed.  Only military, not representative of entire mTBI population.  Cross-sectional design.  No pre-injury scan.  Small portion of female participants. |
| ^3^ | Military  127 veterans | Blasts | Not available  (33 y, 117:10) | 1-5 years post-injury | Resting-state fMRI  3T | Greater blast mTBI severity scores were associated with lower functional connectivity between:  - lateral geniculate nucleus seed and medial frontal gyrus, lingual gyrus, and  right ventral anterior nucleus of thalamus;  - primary visual cortex seed and precuneus;  - lateral occipital gyrus seed and middle and superior frontal gyri;  - fusiform gyrus seed and superior  and medial frontal gyrus and left middle frontal gyrus.  Blast mTBI severity score alone significantly accounted for differences in functional connectivity for the lateral geniculate nucleus, lateral occipital, and fusiform gyrus networks  The number of blast event exposures, number of mTBIs, and education did not explain a significant amount of the variation in functional connectivity when included in the regression model alongside blast-related mTBI severity. | Lower functional connectivity between visual network regions and frontal cortical regions predicted worse performance on the WAIS digit-symbol coding task.  Stroop interference score did not significantly correlate with functional connectivity in the lateral geniculate nucleus, lateral occipital, and fusiform gyrus networks.  No significant correlations between functional connectivity and non-blast mTBI severity scores, intelligence quotient, PTSD diagnoses, alcohol dependence, major depressive disorder, or any axis I disorder, deployment measures (i.e. number of deployments, duration of deployments, time since most recent deployment), or time since most recent blast exposure. | No control group.  Cross sectional design.  Acute-stage injury characteristics were obtained through retrospective self-report, external records and corroborating sources were not available for review, inconsistencies in self-reported combat-related mTBI were observed over time.  Participants may have difficulty accurately representing previous blast and combat events.  Subjects were not monitored with periodic response or eye-tracking during the scan, unable to tell whether participants stayed awake during resting state task.  The study did not include an additional scan with eyes open fixating on a cross.  Abnormalities during rest were not probed while performing a visual task.  Only military, not representative of entire mTBI population.  Tasks not completed inside the scanner. |
| ^4^ | Military active service members  186 mTBI blast  80 mTBI non-blast  21 controls | Blasts and non-blasts, falls, motor vehicle accidents, blunt force trauma, sports, and others | Not available  (33 y, 251:15) | 3 months post-injury | Resting-state fMRI  3T | Blast mTBI group (relative to controls and non-blast mTBI groups) had significant hyper-connectivity.  Blast mTBI group had significantly higher values relative to the control and non-blast mTBI groups in measures describing local structure and information traffic capacity (mean cluster coefficient, efficiency, density, and degrees), in the right and left thalamus.  Blast mTBI group had lower values on measures describing inter-connectivity of nodes (characteristic path length and diameter) relative to the control and non-blast mTBI groups. | Although the blast mTBI group did not display significant differences from controls after controlling for post-traumatic stress symptoms (PTSS), the non-blast mTBI group exhibited significant hypo-connectivity. | Cross-sectional design  Relatively small sample of controls.  Individuals had pharmacological treatment. No washout period described.  Characteristics of blast-related mTBI (distance and intensity of blast exposure) was not reported.  Retrospective, self-reported blast history.  No structured clinical assessment of PTSD. |
| ^5^ | Military  287 chronic mTBI with  low (n=58), medium (n=124),  high (n=105) PTSS  44 civilian controls | Not available | Not available  (35 y, 278:9) | 4 years post-injury | Resting-state fMRI  3T | Connectivity in the medial frontal gyrus  was higher in the control group compared to each of the PTSS mTBI groups. | The effects of PTSS (all mTBI groups vs controls) were noted in the anterior cingulate and parahippocampus.  Evidence of distinct connectivity patterns between each of the PTSS mTBI groups when compared to the control group, as follows:  - regions of the left parahippocampus had higher connectivity within the high PTSS mTBI group in contrast to the low PTSS mTBI group.  - low PTSS mTBI group had higher connectivity in the right middle temporal gyrus and left middle occipital gyrus relative to the medium PTSS mTBI group.  - higher connectivity in the right medial frontal gyrus and left inferior frontal gyrus in the medium PTSS group relative to the high PTSS mTBI group.  - higher connectivity in the right middle frontal gyrus in the high PTSS group relative to the medium PTSS group. | Participants were recruited from a clinical population with heterogeneous symptoms (i.e. pain, sleep disruption).  Regulation of default mode network activity may have been affected by sleep disruption.  Subjects were receiving ongoing clinical care  for injuries in addition to the TBI and were prescribed medications. No washout period described and effects of medications not controlled for.  Controls had a higher number of female participants and some  civilians.  Imaging results from pairwise group comparison t-tests were not corrected for multiple-comparisons. |
| ^6^ | Athletes  92 athletes with sport-related concussion  82 non-injured control athletes | Sport-related concussion | Not available  (19 y, 78:14) | 24-48 h post-injury, following clearance to begin the return-to-play  7 days following unrestricted return-to-play | Resting-state fMRI across 3 sites  3T | No significant between-group differences in regional homogeneity, fractional amplitude of low -frequency fluctuation, or  average nodal strength. This indicates that concussion did not result in abnormalities in local connectivity (regional homogeneity), the relative amplitude of slow oscillations (low -frequency fluctuation) or global connectivity strength (average nodal strength) beyond the clinical recovery period.  No group differences in subject-specific abnormalities including the number and volume of low-frequency fluctuation or regional homogeneity clusters.  Concussed athletes had elevated regional homogeneity in the right middle and superior frontal gyri at the 24-h visit which returned to normal levels at the following visit. | Concussed athletes with greater regional homogeneity in the middle and superior frontal gyri at 24-h post-injury had significantly greater psychological symptoms (e.g. mood dysfunction) at subsequent visit (on average 10.43 days post-concussion).  No significant association between regional homogeneity and clinical measures (including symptom severity, psychological symptoms, postural stability and cognition) at 24-h.  No significant association between regional homogeneity and the number of days until athletes were asymptomatic. | Attrition rate unclear.  Number of female athletes included in the study was relatively small.  This study only  included athletes that ultimately recovered from their injury. Results may not generalize to other samples that show persistent concussion symptoms.  Scanner and sequence variation across sites. |
| ^7^ | Civilians  97 acute mTBI  56 chronic mTBI  43 healthy controls | Not available | Acute mTBI (39 y, 48:49)  Chronic mTBI (35 y, 33:23) | 7 days post-injury  6 months to 1 year post-injury | Resting-state fMRI  3T | Acute mTBI patients, compared to controls, had enhanced functional connectivity between the inferior fronto-occipital fasciculus white matter functional network and primary sensorimotor white matter functional networks, as well as cortical primary sensorimotor grey matter functional networks.  Abovementioned alterations in white matter functional network returned to the baseline level at the chronic stage. | Functional connectivity between inferior fronto-occipital fasciculus white matter functional network and anterior cerebellar grey matter functional network positively correlated with information processing speed, as measured via the Trail-Making Test Part A. | White matter signals might have infiltrated from the grey matter causing partial volume effect.  Data not collected beyond one year.  Type of injury not specified. |
| ^8^ | Civilians  105 mTBI participants of which:  44 chronic pain group  61 recovery group | Motor vehicle collision | Chronic pain group: [25-75%] age range 28-44 y  (37 y, 19:25)  Recovery group: [25-75%] age range 27-41 y  (35 y, 36:25). | 72 hours post-injury | Resting-state fMRI  3T | Reduced negative functional connectivity between nucleus accumbens and primary motor cortex in chronic pain group.  Complementary pattern between periaqueductal gray matter and primary somatosensory cortex.  Increased functional connectivity between periaqueductal gray matter, nucleus accumbens, and rostral anterior cingulate cortex in recovery group.  Functional connectivity features shortly after mTBI predict transition to long-term chronic pain. | Not available. | No controls.  Cross sectional design.  Some clinical outcome measures were missing, hence patients not fully phenotyped. |
| ^9^ | Not available  Dataset 1: 179 scans of which 41 healthy, 93 mild-TBI and 45 severe-TBI  Dataset 2: 10 healthy  Dataset 3: 198 healthy  Dataset 4: 21 healthy | Not available | Not available  mTBI (45 y, 49:33)  severe TBI (35 y, 9:9) | Not available | Resting-state fMRI  3T | Tested a deep learning approach based on a Siamese Network to learn a discriminative  feature representation for single-subject independent component analysis classification.  Siamese independent component analysis is robust to scan-rescan variation, and shown to outperform traditional convolutional neural network and  template matching methods in identifying eleven subject-specific functional resting state networks, achieving 100%  accuracy on a holdout data set and over 99% accuracy on an outside data set.  The functional connectivity of default mode and salience networks identified by the  proposed technique is altered in a group analysis of mTBI,  severe TBI, and healthy subjects. | Not available. | Four individual datasets are used for training and evaluating the proposed model, and only one dataset has participant with TBI. Results may be confounded by site effects and variances. |
| ^10^ | Civilians  88 mTBI  85 healthy controls | Not available | 18-60 y  (40 y, 43:45) | 3 days post-injury | Resting-state fMRI  3T | All groups showed significant rich-club organization.  The rich-club  organization was aberrant, with enhanced functional connectivity among rich-club nodes and peripheral  regions in acute mTBI.  Significant differences in partial global and local network topological  property measures were found between mTBI patients and controls. | Not available. | An important limitation of graph theory analysis is the arbitrary threshold and binarization process,  which can lead to information loss.  No information on type of injury. |
| ^11^ | Civilians  109 acute mTBI, of which 41 followed up in chronic phase  65 healthy controls | Road traffic accident, falls, violence or assault, other | Not available  Acute mTBI:  (39 y, 61:48)  Chronic mTBI: (32 y, 23:18) | Acute: 1-week post-injury  Chronic follow-up: 6-12 months | Resting-state fMRI  3T | Dynamic cross-network interactions were significantly increased and more variable in mTBI compared to controls.  mTBI exhibited an increased integration between the salience network and central executive network while reduced coupling of the salience network with default mode network. | Increased network interaction index in mTBI implies more severe and multiple cognitive impairments. | Patient attrition rate may have biased the sample.  Limited cognitive domain measures conducted in mTBI patients. |
| ^12^ | Civilians  104 acute mTBI, of which 41 followed up in chronic phase  56 healthy controls | Car accident, fall, assault, drop, other mechanism | Not available  (38 y, 54:50) | Acute phase: 7 days post-injury | Resting-state fMRI  T1w  3T | Altered structural and functional connectivity  coupling of the sensorimotor network in acute mTBI.  Persistent structural and functional connectivity decoupling of the sensorimotor network and additional decoupling of the default mode network in chronic mTBI. | Decoupling of sensorimotor network and default mode network predicts better cognitive outcomes. | Attrition of patients in the chronic phase.  Relatively small sample size of cognitive subgroups in the chronic phase. |
| ^13^ | Civilians  108 mTBI, of which 31 patients scanned longitudinally  76 healthy controls | Road traffic accidents, incidental fall, other non-intentional injury, violence/assault, act of mass violence, unknow | 18-70 y  (not available, 69:39) | 14 days post-injury  6 months  12 months | Resting-state fMRI  T1w  3T | No significant structural  changes.  Thalamic hyperconnectivity was found in mTBI in acute phase.  Acute fMRI markers (i.e., acute thalamic hyperconnectivity in mTBI, with specific vulnerabilities of individual thalamic nuclei) differentiated those with chronic post-concussive symptoms.  No significant differences between GOSE (‘complete’ [GOSE-8]versus ‘incomplete’ [GOSE ≤7] recovery), and PCS groups (post-concussive symptom-[PCS] positive or negative) in blood biomarkers, including neuron-specific enolase (NSE), S-100 calcium-binding protein B (S100B), glial fibrillary acidic protein (GFAP), Tau, ubiquitin C-terminal hydrolase-L1 (UCH-L1) and neurofilament light chain (NFL). | Emotional and cognitive symptoms were  associated with changes in thalamic functional connectivity. | The thalamus and its subdivisions were not individually defined in each patient.  Prevalence rates of post-concussive  symptom in mTBI populations varied depending on the classification  method used.  Blood-based biomarker levels vary substantially  over time post-injury. |

Supplementary Table 3. Studies assessing mild traumatic brain injury (mTBI) utilising functional magnetic resonance imaging (fMRI). Other abbreviations: post-traumatic stress disorder (PTSD), post-traumatic stress symptoms (PTSS), T_1_-weighted (T1w)

1. Robinson ME, Lindemer ER, Fonda JR, Milberg WP, McGlinchey RE, Salat DH. Close-range blast exposure is associated with altered functional connectivity in Veterans independent of concussion symptoms at time of exposure. *Hum Brain Mapp*. Mar 2015;36(3):911-22. doi:10.1002/hbm.22675

2. Spielberg JM, McGlinchey RE, Milberg WP, Salat DH. Brain network disturbance related to posttraumatic stress and traumatic brain injury in veterans. *Biol Psychiatry*. Aug 1 2015;78(3):210-6. doi:10.1016/j.biopsych.2015.02.013

3. Gilmore CS, Camchong J, Davenport ND, et al. Deficits in Visual System Functional Connectivity after Blast-Related Mild TBI are Associated with Injury Severity and Executive Dysfunction. *Brain Behav*. May 2016;6(5):e00454. doi:10.1002/brb3.454

4. Nathan DE, Bellgowan JF, Oakes TR, et al. Assessing Quantitative Changes in Intrinsic Thalamic Networks in Blast and Nonblast Mild Traumatic Brain Injury: Implications for Mechanisms of Injury. *Brain Connect*. Jun 2016;6(5):389-402. doi:10.1089/brain.2015.0403

5. Nathan DE, Bellgowan JAF, French LM, et al. Assessing the Impact of Post-Traumatic Stress Symptoms on the Resting-State Default Mode Network in a Military Chronic Mild Traumatic Brain Injury Sample. *Brain Connect*. May 2017;7(4):236-249. doi:10.1089/brain.2016.0433

6. Meier TB, Giraldo-Chica M, España LY, et al. Resting-State fMRI Metrics in Acute Sport-Related Concussion and Their Association with Clinical Recovery: A Study from the NCAA-DOD CARE Consortium. *J Neurotrauma*. Jan 1 2020;37(1):152-162. doi:10.1089/neu.2019.6471

7. Jia X, Chang X, Bai L, et al. A Longitudinal Study of White Matter Functional Network in Mild Traumatic Brain Injury. *J Neurotrauma*. Oct 1 2021;38(19):2686-2697. doi:10.1089/neu.2021.0017

8. Bosak N, Branco P, Kuperman P, et al. Brain Connectivity Predicts Chronic Pain in Acute Mild Traumatic Brain Injury. *Ann Neurol*. Nov 2022;92(5):819-833. doi:10.1002/ana.26463

9. Chou Y, Chang C, Remedios SW, Butman JA, Chan L, Pham DL. Automated Classification of Resting-State fMRI ICA Components Using a Deep Siamese Network. *Front Neurosci*. 2022;16:768634. doi:10.3389/fnins.2022.768634

10. Li F, Liu Y, Lu L, et al. Rich-club reorganization of functional brain networks in acute mild traumatic brain injury with cognitive impairment. *Quant Imaging Med Surg*. Jul 2022;12(7):3932-3946. doi:10.21037/qims-21-915

11. Li X, Jia X, Liu Y, et al. Brain dynamics in triple-network interactions and its relation to multiple cognitive impairments in mild traumatic brain injury. *Cereb Cortex*. May 24 2023;33(11):6620-6632. doi:10.1093/cercor/bhac529

12. Pan Y, Li X, Liu Y, et al. Hierarchical brain structural-functional coupling associated with cognitive impairments in mild traumatic brain injury. *Cereb Cortex*. Jun 8 2023;33(12):7477-7488. doi:10.1093/cercor/bhad053

13. Woodrow RE, Winzeck S, Luppi AI, et al. Acute thalamic connectivity precedes chronic post-concussive symptoms in mild traumatic brain injury. *Brain*. Aug 1 2023;146(8):3484-3499. doi:10.1093/brain/awad056
